# Mpox disease, diagnosis, and point of care platforms

**DOI:** 10.1002/btm2.10733

**Published:** 2025-01-02

**Authors:** Nazente Atceken, Ikra Bayaki, Berk Can, Defne Yigci, Savas Tasoglu

**Affiliations:** ^1^ School of Biomedical Sciences and Engineering Koç University Istanbul Turkey; ^2^ Koç University Translational Medicine Research Center (KUTTAM), Koç University Istanbul Turkey; ^3^ School of Medicine Koç University Istanbul Turkey; ^4^ Department of Molecular Biology and Genetics, Faculty of Science and Letters Yıldız Technical University Istanbul Turkey; ^5^ Institute for Cell Biology University of Bonn Bonn Germany; ^6^ Department of Mechanical Engineering Koç University Istanbul Turkey; ^7^ Koç University Is Bank Artificial Intelligence Lab (KUIS AI Lab) Koç University Istanbul Turkey; ^8^ Koç University Arçelik Research Center for Creative Industries (KUAR) Koç University Istanbul Turkey; ^9^ Boğaziçi Institute of Biomedical Engineering Boğaziçi University Istanbul Turkey

**Keywords:** loop‐mediated isothermal amplification (LAMP), Mpox (MPX), point‐of‐care (PoC) applications, polymerase chain reaction (PCR), recombinase polymerase amplification (RPA)

## Abstract

Human Mpox disease (MPX) is an endemic zoonotic disease that develops when patients are infected with the Mpox virus (MPXV). MPXV shares a high level of genetic similarity to other poxviruses and the clinical presentation of MPX is similar to other poxvirus infections which can result in a delay in diagnosis. In addition, the MPXV virus is phylogenetically divided into two different clades which affects the severity of disease. In recent years, there has been an unusual worldwide spread of MPXV, leading to a global public health problem. The most important step in the fight against MPX is rapid, highly specific, and accurate diagnosis. Following the rapid spread of disease in recent years, efforts to develop diagnostic tests have gained momentum. Here, MPX, MPX epidemiology, and MPX diagnostic tests are discussed. Furthermore, biochemical diagnostic tests, molecular diagnostic tests and their development, and point‐of‐care (PoC) diagnostic applications are reviewed. Molecular diagnostic technologies such as polymerase chain reaction, recombinase polymerase amplification, and loop‐mediated isothermal amplification methods that detect MPX are evaluated. Additionally, next‐generation combined molecular techniques and their importance in PoC transition are explored.

**Translational Impact Statement**

This review provides a comprehensive evaluation of diagnostic approaches for human Mpox disease (MPX), highlighting the critical need for rapid and precise detection methods amidst the recent global spread of Mpox virus (MPXV). By examining a range of diagnostic techniques, including biochemical, molecular, and point‐of‐care methods, the review underscores the importance of advanced diagnostic technologies like polymerase chain reaction, recombinase polymerase amplification, and loop‐mediated isothermal amplification. These insights aim to inform and guide the development of more effective diagnostic tools, which are essential for timely diagnosis, effective disease management, and improved public health responses. The review's findings offer valuable guidance for enhancing diagnostic capabilities and addressing the global health challenge posed by MPX, with potential implications for similar infectious diseases.

## 1 INTRODUCTION

Human Mpox disease (MPX) is an infectious disease caused by the Mpox virus (MPXV), a DNA virus from the family Orthopoxviridae.^1^ There are two subclasses of the MPXV virus. Clade I is defined as having higher virulence but less transmissibility, while Clade II has lower virulence and is characterized by a faster spread.^2^ Endemic in wild rodents in Central and West Africa, MPX is considered a pathogenic agent that can infect humans and some primates and can cause serious health problems.^3^ Human‐to‐human transmission was first reported in 1970, then the number of cases increased in Central and West African countries, where the virus is considered to be endemic.^4^ After the COVID‐19 outbreak, a case of MPX was observed in the United Kingdom in 2020; there was a rapid spread, and MPX was declared a Public Health Emergency of International Concern by the World Health Organization (WHO) in July 2022.^4^, ^5^ Although it has been endemic in some parts of Africa for many years and is rare in humans, its worldwide spread and incidence have increased in recent years.^5^ The routes of transmission between animals and humans include direct skin contact and mucosal exposure while human‐to‐human transmission occurs through respiratory secretions or debris.^3^, ^6^ The incubation period of the disease is usually between 7 and 17 days, and a patient may be contagious before symptoms become apparent. Symptoms of human mpox include high fever, headache, muscle and joint pain, fatigue, lymphadenopathy, and rashes. The rash first begins on the face and hands and can then spread to other parts of the body.^6^ These symptoms play an important role in the clinical diagnosis of the disease. MPXV exhibits clinical and phylogenetic similarities to viruses from the same family.^6^ Therefore, the most important element in controlling the spread of disease is achieving early and accurate diagnosis of MPX.

Clinical evaluation, laboratory tests and imaging techniques are used in the diagnosis of the disease. Clinical evaluation involves making a preliminary diagnosis based on the patient's symptoms. Laboratory tests allow samples taken from the rash to be examined and confirm the presence of MPX virus. Imaging techniques can be used when necessary. Although some clinical features such as lymphadenopathy can help differentiate MPX from other poxvirus infections, many of the prodromal clinical symptoms are similar to *Orthopoxviruses*, and therefore, observation of clinical symptoms is insufficient for diagnosing mpox.^2^, ^7^ Therefore, in addition to clinical symptoms, MPX diagnosis can be made using laboratory‐based nucleic acid amplification tests (NAATs) and by targeting gene sequences specific to MPXV DNA.^2^, ^8^ Among laboratory‐based NAATs, polymerase chain reaction (PCR) is currently the most widely used diagnostic tool and is recommended by the WHO owing to its high specificity and sensitivity.^4^, ^9^ Apart from qPCR, immunological tests, and imaging with electron microscopy by negatively staining viral particles are also employed.^5^, ^8^ However, all of these methods require expert personnel and extensive and complex laboratories. Laboratory facilities are very limited, especially in the African region where the virulence and prevalence of the virus are high.^8^ Therefore, it is essential to develop rapid and highly sensitive point‐of‐care diagnostic (PoC) systems that can be applied in the field without the need for experts and extensive laboratory facilities. MPX is a disease that can lead to severe consequences including the development of pneumonia, encephalitis, and septicemia and carries potential risks of spreading.^4^, ^10^ It is therefore important to continuously support scientific research and public health measures to recognize, treat, and prevent the spread of the disease. This approach may be an effective strategy in controlling the disease. With the COVID‐19 pandemic, the importance of fast, accessible and highly accurate diagnostic tests in preventing and controlling the spread of infectious diseases has been emphasized.^11^ In this context, it is very important to develop high‐accuracy PoC diagnostic systems.^12^

Here, MPXV, its epidemiology, and clinical symptoms are summarized. The methods currently used for diagnosis along with their shortcomings and advantages are discussed. PoC and rapid diagnostic platforms that have been developed to control the spread of viral epidemics, the methods used to develop these platforms, the strategies and diagnostic platforms used for the diagnosis of MPXV are reviewed.

## 2 MPOX DISEASE

MPXV is a zoonotic virus that wraps its double‐stranded DNA (dsDNA) with a lipoprotein envelope and belongs to the *Orthopoxvirus* genus of the Poxviridae family. Among the viruses of the *Orthopoxvirus* genus, are *Variola* virus and *Cowpox* virus, which cause smallpox and cowpox, respectively. *Vaccinia* virus, which is used in the production of smallpox vaccine also belongs to the *Orthopoxvirus* genus.^13^, ^14^, ^15^

MPXV has the same morphological features as other viruses of the *Orthopoxvirus* genus. Poxviruses that belong to the *Poxvridae* family are viruses with an oval structure with dimensions of 200–400 nm under the electron microscope. Similarly, MPXV virus is in the 200–250 nm size range.^16^ It contains dsDNA (≈197 kb), protected by the outer membrane, transcription factors, and enzymes.^17^ The MPXV dsDNA genome consists of palindromic hairpins, tandem repeats, inverted terminal repeats (ITRs), and open reading frames (ORFs) at its ends, but some of the ORFs are incomplete or truncated unlike other orthopoxviruses. The MPXV virus, which carries out its entire life cycle in the cytoplasm of the infected cell, encodes all the proteins necessary for viral DNA replication, transcription, and exit from the cell with its genome. MPXV replication is complex but is thought to be similar to that of other orthopoxviruses.^6^, ^16^, ^18^

When genetic, clinical, and geographical differences are evaluated, the MPXV consists of two main clades. These clades are classified into Clade I (formerly known as the Congo Basin strain) and Clade II (formerly known as the West African strain). Clade I causes more severe infection and higher mortality rates, while the Clade II is weaker.^14^, ^15^ There is a geographical separation between the two clades. To date, Cameroon is the only country where both viruses have been found.^14^ In the event of a global spread, the geographical boundaries of these two clades may change. Until recently, it was considered an infectious disease endemic to West and Central Africa. However, in the last few years, the disease has become more widespread and has been observed globally in patients who have not traveled to MPX‐endemic regions. Therefore, it is important to distinguish between the two clades of MPXV to predict patient prognosis.

### 2.1 Mpox epidemiology

MPXV was first observed in colonies of cynomolgus monkeys kept for research in Denmark and was recorded as a disease outbreak when monkeys showed smallpox‐like symptoms.^19^ The first human case of MPXV was reported in 1970 in the Democratic Republic of Congo, a West African country, and subsequently identified as a human pathogen.^20^ It was determined that human MPX cases increased in West and Central Africa between 1970 and 1979, and 21% of the cases detected in the Democratic Republic of the Congo resulted in death. In 1980, the WHO funded and supported virus and disease studies in the Congo Basin area.^21^, ^22^ In 2003, it was confirmed that the MPXV outbreak in the United States was caused by gnawers imported from Africa.^23^ This was the first recorded outbreak to occur outside of Africa.^6^ Between 1971 and 1978, three cases of MPXV were recorded in Nigeria, with no laboratory‐confirmed fatal cases. Nigeria, which has not reported any MPXV cases for 39 years, reported laboratory‐confirmed cases from 17 states to WHO in 2017. Of the 68 confirmed cases, 2 resulted in death. As of 2019, cases have been reported from different regions of Nigeria.^16^ In Nigeria's 2017–2019 MPXV outbreak, 424 suspected cases and 155 confirmed cases were reported. Individuals in the 21–40 age group were most affected by this outbreak. Additionally, among confirmed cases, 75% were observed in male individuals. Due to the more critical importance of the COVID‐19 pandemic in 2020, Nigeria used its resources for the new global outbreak and therefore there were no recorded cases of MPXV in 2020. The reasons for the increase in reports of MPXV cases in Nigeria between 2017 and 2019 include the lack of smallpox vaccination for a large part of the population, close contact with nonhuman primates, especially rodents and wild mammals, human violation of wildlife, increased trade in rodents and other wild animals, and consumption of animal flesh. The incidence and reporting of cases in Nigeria during these years suggested that MPX was an endemic disease.^24^

Until recently, it was considered an infectious disease endemic to West and Central Africa. However, over the previous few years, the disease has become more widespread than in previous periods. It has been observed in people from different regions with no direct contact to endemic regions. From January 2022 to October 2023, cases were reported from 116 countries; the total number of cases was 91,788; and the number of cases resulting in death was 167. Most cases have been reported in the United States.^25^ In May 2022, the virus began to spread between continents after a patient with skin lesions traveled from Nigeria to the United Kingdom. qPCR testing enabled the detection of Clade II of the MPXV virus. While the source of the infection has not been determined, the virus then spread to many European countries. About a month after the first case was observed in the United Kingdom, 92 confirmed cases were reported to the WHO from 12 countries where the virus is not endemic, such as Australia, Canada, Belgium, France, Germany, the Netherlands, Sweden, Spain, Portugal, the United States, and Italy. None of these cases have reported traveling to endemic areas. For this reason, the source of the epidemic has not yet been determined. A genome sample from a confirmed case showed a close match to cases observed in Nigeria between 2017 and 2019.^26^, ^27^

WHO declared the ongoing MPX outbreak a “Public Health Emergency of International Concern” in the summer of 2022.^6^ According to WHO data updated in November 2023, in October 2023, 668 new cases were detected in 29 countries and confirmed by the WHO. The regions with the highest transmission are not Mpox‐endemic; they have been reported from the Western Pacific and European regions. Eight laboratory‐confirmed cases have been reported from the African region where the virus is endemic.^25^ Epidemiological research is still ongoing in the Democratic Republic of Congo where the highest number of cases annually has been reported. From the beginning of 2023 until November, 12,569 cases were reported to WHO from the Democratic Republic of Congo, 581 of which resulted in death. In WHO's November 2023 report, sexual transmission of Clade I MPXV was reported for the first time. The possibility of rapid spread via sexual transmission has raised concerns at national and international levels. MPXV remains endemic in Africa, especially in the Democratic Republic of Congo.^25^ Between October 1 and 31, 29 countries reported cases, with Germany reporting the highest increase in the European Region and Vietnam in the Western Pacific Region. The United States, Brazil, Spain, France, Colombia, Mexico, the United Kingdom, Peru, Germany, and China were the countries reporting the highest cumulative number of cases as of the end of October 2023. Cases reported from these countries constitute 81.7% of the cases worldwide.^25^

**FIGURE 1:**
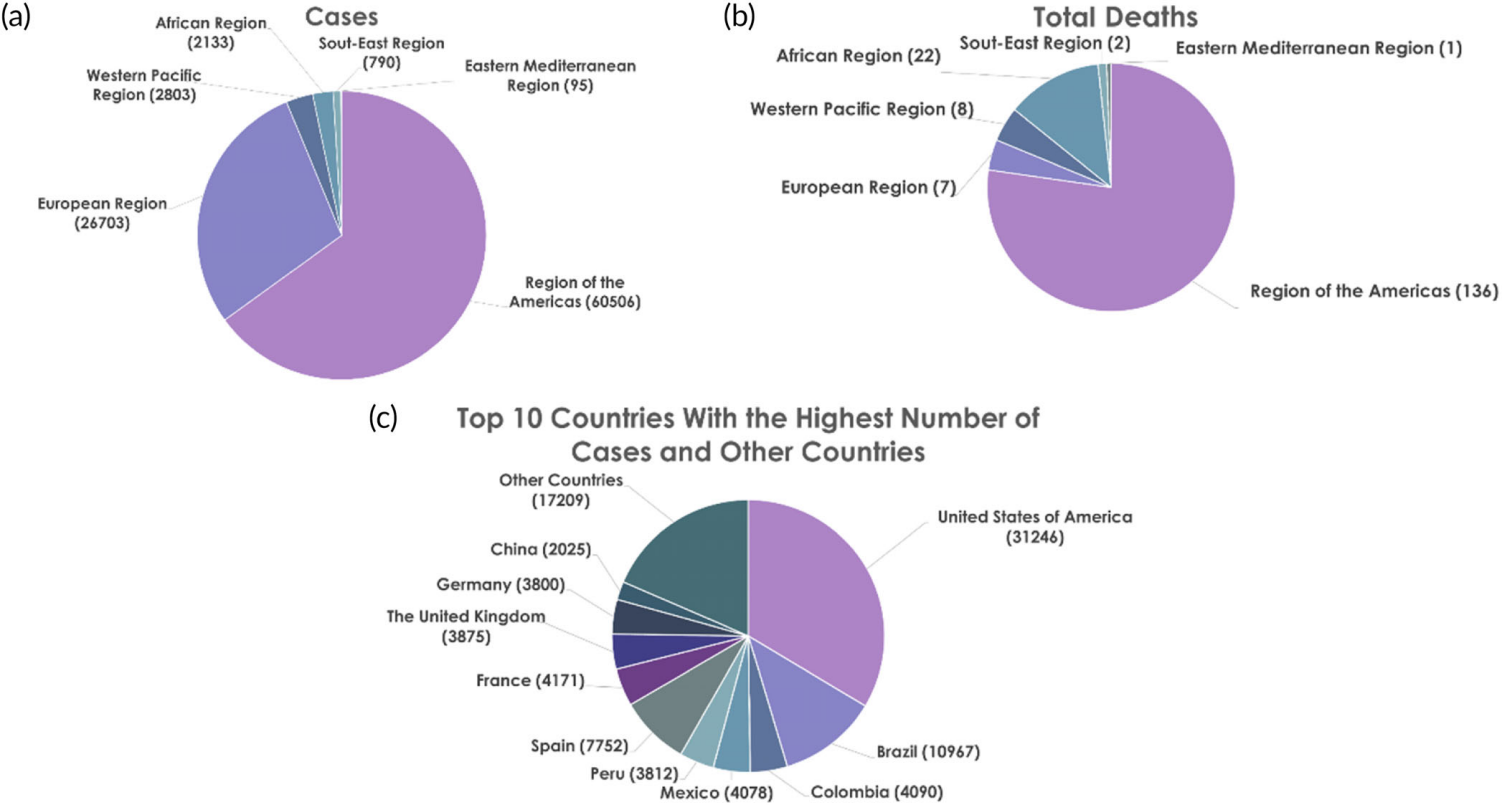
Chart representation of World Health Organization (WHO)‐verified Mpox data from September 2022 to January 2024. Data presented in the WHO January 2024 report were obtained from the CSV.^28^ (a) According to January 2024 data, the total number of cases reported in WHO regions are expressed on the graph. (b) Total number of confirmed deaths across WHO regions, based on January 2024 data. (c) Case numbers of the top 10 countries reporting the most cases to WHO and the other 107 countries from September 2022 to January 2024.

When the general MPXV case profile was examined according to January 2024 data, 96.4% of current cases were observed in male individuals in the 18–44 age group (average age: 34). Men in this age group continue to influence the course of the outbreak. 3.6% of the cases are female, and the majority of female cases are reported from the Americas and Europe. According to the data, the most common form of transmission is sexual transmission with a rate of 83.2%. Transmission has been reported largely in men who have sex with men (85.3%). While 1.3% of the cases are in the 0–17 age group, 0.4% of these are children in the 0–4 age group. 99% of the cases reported from July 2023 to December 2023 were men and the most common form of transmission was reported through sexual contact with 97.7%. The number of cases and deaths in WHO regions according to January 2024 data are shown in Figure 1. In this report, Cambodia reported a case for the first time, and MPXV cases have been reported from a total of 117 countries until now. The number of confirmed cases is 93,030 and 176 of these cases have been reported as dead.^28^ While the mortality rate is relatively low, MPX poses a global health risk that should not be underestimated.

Latest data portraying the epidemiology, spread of the virus in non‐endemic countries, the route and rate of transmission, and transmission rates by gender and age support the fact that the virus still poses a global risk. In addition, Clade I which is associated with a higher mortality rate and is more common in endemic regions than Clade II, also poses a risk for spread to non‐endemic regions. As emphasized in the WHO report, it is essential to prevent and slow down the spread of infection globally and nationally.^29^ Health strategies need to be developed for the protection, prevention, and treatment of the spread of the disease. The first step in applying these strategies is a rapid, specific, and accurate diagnosis. Thus, it is necessary to develop POC diagnostic platforms that are simple to use and can be used in the field without the need for expensive equipment in regions where laboratory facilities are limited.

### 2.2 Transmission pattern

**FIGURE 2:**
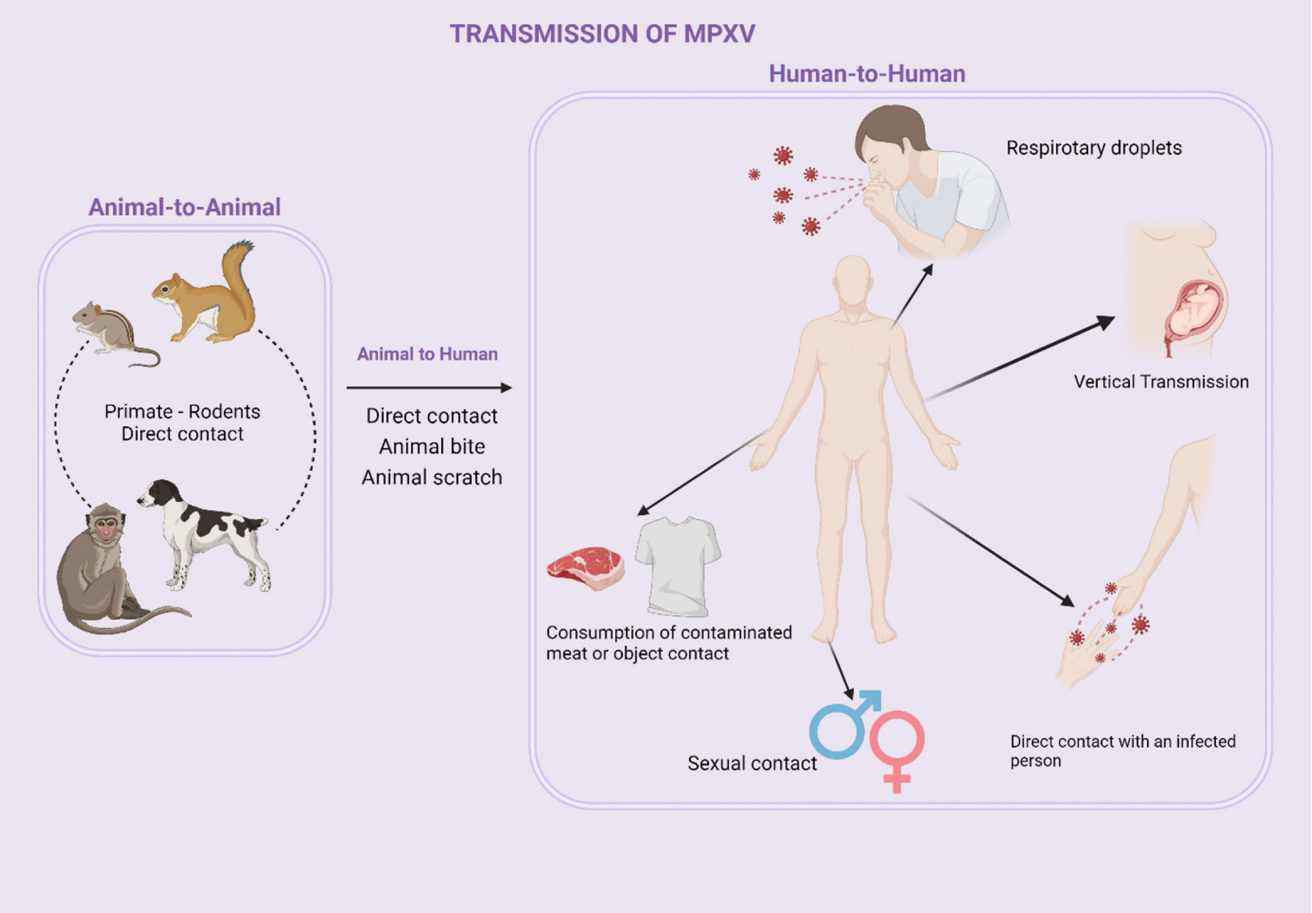
Schematic representation of Mpox transmission pattern. Transmission is divided into three: animal to animal, animal to human, and human to human. Human‐to‐human transmission is divided into two: vertical (transmission from infected mother to baby) and horizontal transmission. Transmission from animal to human occurs through direct contact with infected rodents, or through animal bites and scratches. Horizontal transmission from person to person occurs through sexual contact, close contact with respiratory secretions of the infected individual, contact with contaminated objects, and consumption of infected animal meat.

Although the exact reservoirs of the virus are unknown, animal‐to‐animal transmission occurs. MPXV virus has been isolated from various rodents in Africa, non‐primate animals such as rope squirrels, tree squirrels, Gambian marsupials, dormice, and monkeys, including giant anteaters, and these animals have been proven to be hosts of the MPXV virus. The virus was also isolated from sooty mangabey (*Cercocebus atys*), and MPXV‐specific antibodies were observed in African brush‐tailed hedgehogs. MPXV has been detected in many rodents imported from Ghana to the United States, such as Gambian opossums, rope squirrels, and African dormice, and has been transmitted to prairie dogs in the same area as a result of animal‐to‐animal transmission. Animal‐to‐human transmission occurred as a result of the importation of infected prairie dogs into the United States, and human‐to‐human transmission resulted in the 2003 US outbreak^15^, ^26^, ^30^ (Figure 2).

MPXV is transmitted from animals to humans through direct contact, such as through contact with the blood of infected animals, bites, scratches, or consumption of infected animal flesh. In the first outbreak in the United States in 2003, 47 cases were proven to be caused by close contact with domestic prairie dogs (*Cynomys* spp.) kept with rodents imported from Ghana.^26^ Human‐to‐human transmission has been associated with respiratory particles, belongings of the infected person, and close contact, such as contact with skin lesions and body fluids.^16^, ^31^ The virus can be transmitted through sexual contact with an infected individual. A study conducted in Germany found that the infection was mostly transmitted through close contact and sexual interaction. In the study conducted on four patients, predominantly mild clinical symptoms were observed, while fever and lymphadenopathy were observed in two patients. It is thought that severe symptoms are caused by transmission through contact with inflammatory lesions.^32^, ^33^ Although sexual transmission has been proven in research, spread through semen or vaginal fluids is unclear.^26^

In addition to horizontal transmission, spread via vertical transmission has also been reported. Vertical transmission is defined as transmission from mother to fetus during pregnancy or birth. In the clinical research conducted in the Democratic Republic of Congo between 2007 and 2011, fetal death occurred in three out of four infected pregnant women and the pregnancy was terminated while the asymptomatic individual had a healthy birth. Fetal death occurred in one of the individuals due to complications related to MPXV infection in the 18th week of pregnancy. Skin lesions were observed, especially on the soles of the feet and palms of the stillborn fetus. High levels of MPXV were detected in samples taken from fetal tissue, placenta, and umbilical cord using PCR. The virus was also detected in the peritoneal fluid obtained from the fetus with the MPXV‐specific PCR test.^34^, ^35^

### 2.3 Clinical features

Although differences exist, the clinical presentation of MPX is similar to that of smallpox. Upper respiratory tract associated symptoms such as sore throat and cough are commonly reported in early stages of smallpox infection and less commonly observed in MPX. The approximate incubation period of MPX may vary between 1 and 3 weeks and nonspecific prodromal symptoms of fever, fatigue, myalgia, and headache occur in the first 5 days of infection, followed by lymphadenopathy and the eruption of a rash and pleomorphic skin lesions.^24^, ^32^ In many patients, maxillary, cervical, or inguinal lymphadenopathy is observed before and during the onset of skin manifestations which can help distinguish MPX from smallpox. While lymphadenopathy is a common clinical manifestation in MPX, it is rarely reported in smallpox. The lymph nodes in MPX become swollen, hard, tender, and painful in some patients.^36^ The rash typically originates at the face and spreads to the limbs and then to the palms and soles. Progression of skin lesions (macule, papule, vesicle, pustule, scab phases) is often observed.^37^ Lesions are painful until scab formation begins (in approximately 2 weeks). Within 3 days of onset of the skin lesions, fever typically begins to subside. While most patients exhibit skin lesions on the face, palms and soles, and oral mucous membranes, lesion can also be observed in the conjunctiva and genitalia.^38^ Oropharyngeal lesions, mouth ulcers, and tonsillitis are among other clinical symptoms encountered in infected individuals. The disease lasts for 2–4 weeks and the skin lesions heal spontaneously during this time. In some patients, skin erosion occurs after the lesions heal. Secondary bacterial infections on the skin have been observed due to skin erosion. Additionally, gastrointestinal symptoms such as vomiting and diarrhea may occur.^21^, ^26^, ^36^ Severe complications such as pneumonia, encephalitis, and septicemia have been reported.^38^ Relying solely on clinical symptoms such as lymphadenopathy, skin lesions, and fever is not sufficient to rule out differential diagnoses and it is essential to use virus‐specific diagnostic tests make a definitive diagnosis of MPX.

## 3 DIAGNOSIS AND ADVANCED POINT OF CARE PLATFORMS

The development of rapid and accurate diagnostic tools is essential to enable early diagnosis and effective follow‐up of infectious diseases such as human MPX. In addition to traditional laboratory‐based PCR and immunological tests, PoC applications, which have the potential to provide rapid diagnosis in field studies or small health centers, offer a remarkable advantage in this field. Biochemical and molecular techniques can facilitate rapid, accurate, and sensitive diagnosis. However, biochemical techniques are not preferred because they often fall short in achieving high specificity.

Molecular diagnostic methods, on the other hand, allow specific and accurate detection because they focus on genetic sequence differences. Among NAATs, the most commonly used molecular strategy is PCR. In order to employ PCR in PoC applications, isothermal amplification methods recombinase polymerase amplification (RPA) and loop‐mediated isothermal amplification (LAMP) are frequently used. In addition, new‐generation diagnostic methods such as the use of isothermal NAATs in combination with CRISPR has emerged as powerful strategies for PoC applications. CRISPR combined technologies can speed up the diagnostic process by targeting viral RNA or DNA and allow detection of pathogen genome within a few hours.^39^, ^40^ The advantages of these methods include features such as fast results, low costs, and portability. However, these new approaches need to be carefully evaluated in terms of sensitivity, specificity, and usability under field conditions. The importance of PoC applications in the diagnosis of MPXV is of great importance as it can provide rapid recognition of the infection, early initiation of treatment, and control over spread of disease. Research on MPXV diagnosis and PoC applications is an important step for early diagnosis and effective control of infectious diseases. Advances in such fields are expected to facilitate rapid and effective health systems response to disease spread and protect public health.

### 3.1 Biochemical diagnosis

The methods used to detect pathogens in viral infections can be divided into two categories: nucleic acid detection and antigen detection. These two methods serve different purposes and a complementary diagnostic approach.^41^ Although molecular methods are currently used more frequently in the diagnosis of MPXV, serological approaches are used in epidemiological studies, and variant seroprevalence studies are conducted to examine past exposure to MPXV.^42^, ^43^ It is possible to categorize serological approaches used to detect the spread of antibodies formed to respond to the disease during and after MPXV infection into three^42^: the immunofluorescence assay (IFA) is used to detect IgG and IgM; enzyme‐linked immunosorbent assay (ELISA) is designed for use in epidemiological studies; and neutralization assays are used to detect neutralizing antibodies.^43^ In addition to these, methods such as lateral flow immunoassay (LFIA)^44^ and colloidal gold method immunoassay^45^ assay have also been used in some MPXV diagnosis studies.

Immunological methods include techniques such as ELISA for the detection of IgG and IgM antibodies and immunohistochemistry for the detection of viral antigens.^16^ Immunohistochemistry can differentiate between smallpox virus infection and herpes virus using polyclonal or monoclonal antibodies.^16^ It has been observed that antiviral antibodies and T‐cell responses increase at the onset of the disease. IgM and IgG antibodies are detected in serum in approximately 5 and 8 days after the appearance of the rash, respectively. The co‐presence of IgM and IgG antibodies can confirm the diagnosis of MPXV in an individual with severe disease prior to vaccination and a history of rash. However, these methods are not specific for MPX and may indicate the presence of other types of OPXV.^16^, ^46^ IgM can be used to evaluate MPX infection in an individual with a previous history of smallpox vaccination.^47^ A positive IgM capture ELISA indicates that there may have been recent exposure to OPXV in both previously unvaccinated and vaccinated individuals (possibly MPXV in endemic areas), whereas a positive IgG capture ELISA indicates that the individual may have been previously vaccinated or exposed to natural infection.^48^, ^49^ Thus, the presence of both IgM and IgG in a sample is a strong indicator of recent exposure to OPXV in individuals previously vaccinated or exposed to natural infection. Therefore, the presence of IgM in individuals previously vaccinated against smallpox in areas where MPX is endemic is indicative of recent exposure to MPXV. Anti‐OPXV antibodies have been shown to be cross‐reactive against a wide variety of OPXV species, including VACV, CPXV, and MPXV.^43^ MPXV antibodies can be detected in serum approximately 1–2 weeks after the onset of symptoms.^50^ Although the detection of DNA is the gold standard for confirming MPXV infection, detection of MPXV antibodies by serological tests can help physicians determine the response to infection and aid clinical decision‐making.

Seroepidemiological studies rely on the detection of antibodies in the post‐acute phase to determine the overall burden of the disease or to determine the presence and durability of protective antibodies in patients, which is useful particularly for at‐risk populations.^43^ Additionally, antibody‐based antigen detection tests in the form of side‐wave flow tests or rapid tests have been considered for use to detect MPXV infection. In IFA and ELISA, bound antibodies bind to immobilized viral antigens. In IFA, virus antigens are present on infected and fixed cells and are often present on multiwell slides to enable various degrees of serum dilution to be tested simultaneously for antibody determination. Bound antibodies are detected by adding fluorescently coupled human IgG‐ or IgM‐specific secondary antibodies and reading the signals on fluorescence microscopes.^43^ In one study, an improved dual‐signal LFIA sensor was developed for rapid and highly sensitive detection of MPXV *A29L* protein.^44^ This sensor combines a color mode capable of rapid screening of MPXV in resource‐poor areas and a fluorescent mode capable of quantitative detection with high sensitivity in primary care settings. Instead of traditional colored labels and fluorescent materials, the sensitivity of the sensor was increased using SiO2‐Au core double‐QD shell nanocomposites. These nanocomposites have excellent colloidal/optical stability to provide a stable LFIA system. The proposed method was developed to enable easy‐to‐use MPXV detection: result interpretation could be achieved with the naked eye or rapidly measured with a portable commercial fluorescence device.^44^

A commonly used strategy for rapid detection of MPXV antigens and antibodies is the colloidal gold method.^49^ The accuracy of this method has been approved by the UK's medical regulator, the MHRA.^45^ Samples used for the detection of MPXV antigens include skin lesion swabs and lesion crusts. Determination of IgM and IgG antibodies can be helpful in determining the patient's infection status. However, the diagnosis of MPX cannot be made definitively due to cross‐reactivity between orthopoxviruses, which share some common features that are difficult to distinguish using antigen and antibody tests.^45^ Therefore, serological tests can play an important role in epidemiological monitoring of past infections.

In summary, while PCR has been determined as the gold standard for diagnosing MPXV, some studies have advocated for the development of biochemical tests to diagnose MPXV owing to its rapid result generation and inexpensive price. However, some limitations of serological tests have not yet been addressed: serological cross‐reactivity remains an issue and the fact that antibodies remain in the circulation after infection make it challenging to detect acute infection.

### 3.2 Molecular diagnosis

A significant number of studies in search of a rapid and reliable method for the diagnosis of MPXV have been conducted using molecular diagnostic methods. Molecular diagnosis methods are extremely important due to their high sensitivity and ability to detect even low amounts of pathogens. In addition to these advantages, it is possible to rapidly diagnose the disease and subsequently initiate treatment since molecular diagnostic tools have a low sample‐answer time in detecting pathogens.^51^ NAATs are an important approach for pathogen molecular diagnosis. PCR‐based NAATs, which are highly reliable and relatively easy to standardize, have been accepted as the gold standard by WHO. However, the usability of the PCR‐based diagnosis method in low resource settings is limited due to the fact that high equipment needs and trained personnel requirements are often unmet. Various PoC platforms have been developed to overcome these limitations. Isothermal amplification methods such as LAMP and CRISPR systems to create next‐generation CRISPR combined diagnostic methods. Using these strategies, the requirements for high‐tech equipment and qualified personnel can be overcome to some extent as LAMP, RPA, and CRISPR‐based platforms typically do not require thermal cycling steps like PCR. Furthermore, CRISPR‐based platforms can offer rapid, easy‐to use, highly sensitive, highly specific, and programmable pathogen detection with minimal equipment requirements although such platforms involve a higher degree of design complexity compared to traditional methods.^52^ Apart from combined diagnostic studies, methods such as LAMP and RPA have also been used as diagnostic tools on their own which have enabled the development of low‐cost, easy‐to‐use, rapid PoC MPXV detection platforms. However, standalone LAMP‐based platforms can result in false positives. Several strategies such as mathematical modeling or the use of quenched primers have been employed to overcome the false‐positive risk.^53^, ^54^ Similarly, standalone RPA‐based platforms have several limitations including limited reagent stability and specific protocol or storage requirements.^55^ Studies conducted for MPX disease‐clade detection using molecular diagnostic methods are shown in detail in Table 1.

**TABLE 1:** Diagnostic applications on Mpox viruses.

Amplification method	Combined technology	Target	Assay readout	Limit of detection	Readout time	Reference
qPCR	—	*E9L‐NVAR* and *B6R* genes	Fluorescence signal	10 copies	>60 min	56
qPCR	—	*C3L*, *G2R_G*, and *G2R_ WA* genes	Fluorescence signal	40.4/3.5/8.2 copies	>60 min	57
qPCR	—	*F3L* and *G2R* genes	Fluorescence signal	65.6 copies	>60 min	58
qPCR	—	*TNF* and *G2R* genes	Fluorescence signal	—	—	59
RPA	—	*G2R* gene	Fluorescence signal	16 copies	3–10 min	60
RPA	—	*A27L* and *F3L* Genes	Fluorescence signal	10 copies	5–10 min	61
HDA, RPA	—	*F3L* gene	LFT	9.86 copies	35–75 min	62
VF‐RPA	—	*B7R* gene	VF	8.53 copies	5–10 min	63
LAMP	—	*A27L* and *F3L* genes	Turbidimeter naked eye	20 copies	60 min	64
LAMP	—	*ATI* gene	Real‐time turbidity VDR and LFB	5 copies	40–60 min	65
LAMP	—	*D14L* and *ATI* genes	Real‐time turbidity VDR and LFB	5 copies	40–60 min	66
LAMP	—	*ATI* gene	End‐point detection Real‐time monitoring	28.7 copies	30–40 min	67
LAMP	—	*N4R* gene	Fluorescent visual	100 copies	60 min	68
LAMP	—	*ATI* and *D14L* genes	Agarose gel electrophoresis	—	60 min	69
Free	CRISPR‐Cas12a	*F8L* gene	Fluorescence signal/biosensor	59.5 copies	90 min	70
RPA	CRISPR‐Cas12	*D6R*, *E9L*, *N3R*, and *N4R* genes	Fluorescence signal	1–10 copies	30 min	71
RPA	CRISPR‐Cas12	*B2R* gene	Fluorescence signal	1 copy	45 min	72
RPA	CRISPR‐Cas12a	*D14L* gene	Fluorescence signal	5–10 copies	60 min	73
RPA	CRISPR‐Cas12a	*F3L* gene	Fluorescence signal	1 copy	60 min	74
RPA	CRISPR‐Cas12	*B6R* and *F3L* genes	Fluorescence signal	10.6 copies	35 min	75
RPA	CRISPR‐Cas12a	*F3L* gene	Nanopore readout	19 copies	25 min	76
RPA	CRISPR‐Cas12a	*D14L* and *ATI* genes	Fluorescence signal	10 copies	45 min	77
RPA	CRISPR‐Cas12	*B7R* gene	Fluorescence signal	13.5 copies	35 min	78
RPA	CRISPR‐Cas12a	*E9L* gene	Fluorescence signal	1 copy	30 min	79
RPA	CRISPR‐Cas12a	*G2R* gene	Fluorescence signal LFT	100 copies	20–30 min	80
LAMP	CRISPR‐Cas12b	*D14L* and *ATI* genes	AuNP‐LFB Real‐time fluorescence	10 Copies	40–60 min	1

Abbreviations: LFB, lateral flow biosensor; LFT, lateral flow test; VDR, visual reagent; VF, vertical flow strip.

#### 3.2.1 Polymerase chain reaction

Although a growing number of PoC platforms continue to be developed, MPXV detection currently relies on the use of laboratory‐based NAATs.^29^ Detecting MPXV infection using quantitative or conventional PCR (qPCR or PCR), which involves the detection of specific sequences of viral DNA using NAATs, is the most commonly used method.^13^ The WHO has stated that the gold standard detection tool for detecting MPX viral DNA sequences is qPCR or nucleic acid‐based methods based on next‐generation sequencing. qPCR and traditional PCR which are considered the gold standard for infectious disease diagnosis, play an essential role in the clinical monitoring and follow‐up of diseases. Recently, commercial PCR kits have been introduced for the specific detection of OPXV or MPXV.^81^, ^82^ Various researchers have enabled the diagnosis of MPXV by developing PCR protocols that can detect some OPXV and more specifically MPXV. Some of these are PCR protocols that can distinguish the MPXV clades. In PCR assays where direct MPXV detection is not provided, when the OPXV result is positive, an additional MPXV PCR or sequencing can be performed to confirm MPXV infection. Using the genetic sequence data obtained, it is possible to predict the origin of the MPXV clade as well as the disease progression and prognosis.^32^

A group of researchers performed two real‐time PCR assays for laboratory diagnosis of the disease during the US MPXV epidemic in 2003. These assays involve two different OPXV genes, DNA polymerase (E9L) and envelope protein (B6R). The first assay designed, the TaqMan‐based assay (E9L‐NVAR), targeted the OPXV DNA polymerase gene (*E9L*), allowing the detection of Eurasian OPXVs other than Variola. The second assay was a hybridization assay and detected the envelope protein gene (*B6R*) using the MGB EclipseTM probe. This represents a confirmed detection of MPXV.^56^ Confirmed MPXV‐affected human DNA samples were detected using these assays with 100% specificity. It was demonstrated that MPXV diagnosis can be achieved sensitively and reliably by targeting two viral genes.^56^ In another study, a real‐time PCR assay was performed to ensure the specific detection of MPXV. The results obtained were evaluated and validated by comparing them with the FDA‐cleared pan‐Orthopox qPCR assay to measure the performance of the assay. The PKamp™ MPXV qPCR test showed high specificity and sensitivity by targeting the viral *F3L* gene. In the experiment conducted with 20 clinical MPXV samples, a specific and accurate diagnosis of the disease was achieved with a 100% success rate. However, the limit of detection in the study was recorded as 7.2 genome copies/reaction. The test was approved by the NYS Department of Health and was found to be suitable for use in clinical settings.^83^ In another experiment, a Digital PCR protocol was developed to determine the amount of MPXV in different regions of the human body. Researchers explained that the results obtained from this experiment would show possible scenarios about the transmission routes of MPX DNA. To carry out this study, the QIAcuity digital PCR (dPCR) method was used, which can provide absolute and precise measurement of the quantitative values of MPX DNA. A total of 144 biological samples were taken from 26 patients for the MPXV detection and quantification assay performed using this method. The results obtained from the dPCR MPXV DNA detection assay were compared with in‐house qPCR methods. The mating correlation between the two assays was recorded as 95%.^84^

Although the PCR method is considered the primary diagnostic tool for viral DNA detection, it has various limitations including high cost, trained personnel, high technical knowledge, and high‐cost equipment requirements, and long sample‐response time.^56^ As a result, PCR falls short in offering rapid PoC testing particularly for low resource settings. Various next‐generation combined diagnostic device development projects are currently underway to overcome limitations of conventional PCR. Moreover, next‐generation combined detection mechanisms such as CRISPR‐LAMP and CRISPR‐RPA have been introduced to evaluate the accuracy of these new platforms and to assess their use in PoC settings.

#### 3.2.2 Recombinase polymerase amplification

The RPA‐based pathogen detection platforms have emerged in order to overcome limitations of PCR and to develop faster and simpler PoC tools. Unlike PCR, in the RPA detection process, high‐tech equipment is not required; the sample‐response time is short; and the risk of contamination can be reduced because the entire process can be conducted in a single tube.^85^ The working range of the RPA method, unlike PCR, does not require high temperatures. RPA, which can operate at low temperatures such as 22–45°C degrees, has a relatively better tolerance to temperature fluctuations. Owing to this temperature tolerance, results are obtained more easily under various environmental conditions. Moreover, it can be used in microfluidic devices in bedside applications without requiring extensive laboratory equipment. The RPA method can be used alone or in combination with CRISPR and is frequently preferred in new generation combined PoC viral detection tools as it has high sensitivity and selectivity.^80^

For the RPA method, T4 UvaX and bacillus subtilis Pol I enzymes and primers that are designed specifically to target certain regions of the viral genome are used.^86^ The basis of RPA's working principles is the use of recombinant enzymes obtained from various bacteria or fungi found in nature. This recombinant enzyme, which can operate at room temperature can form a polymer structure by tightly binding to the primary DNA. First, the primer recognizes the complementary sequence present in the template DNA. Then, in order to reach the relevant point of the template DNA, the double‐stranded structure of the DNA is destroyed by a single‐stranded DNA binding protein and DNA polymerase. The resulting new DNA chain grows exponentially rapidly as a product of amplification. The relevant gene can be amplified at room temperature in 20–30 min. Thus, the RPA method, which can be performed faster than qPCR and does not require complex equipment, appears to have advantages over PCR particularly at PoC settings.^63^

Since classical RPA requires heavy equipment and involves various manual processes, it is not suitable for PoC approaches. However, recently, studies have been carried out in which chip‐based and paper‐based microfluidics serve as a platform for RPA, replacing the need for heavy equipment. This feature of microfluidics makes digital RPA possible.^87^ In addition, the development of RPA‐based biosensors has facilitated improvements, allowing researchers to obtain fast results with easy result interpretation. Developing such RPA‐based biosensors enables the miniaturization of RPA platforms and can detect pathogens in any setting, enabling viral disease diagnosis outside of laboratory conditions.^88^

**FIGURE 3:**
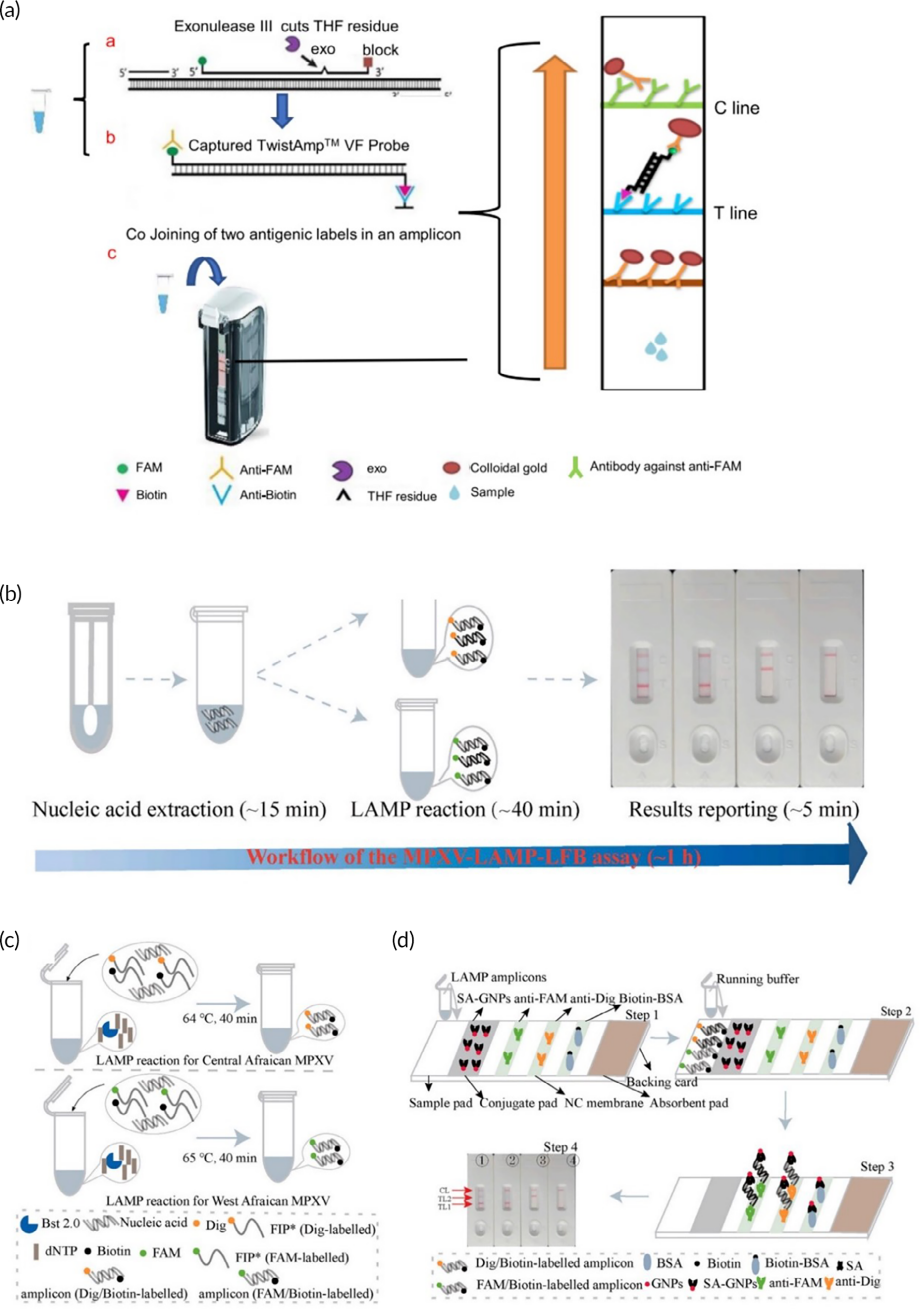
(a) Schematic representation of VF‐RPA MPXV detection platform illustrating the (i) RPA reaction; (ii) VF‐probe hybridization, and (iii) result output which occurs as a result of color development following the detection of nucleic acids. Adapted from Reference [63] under CC BY‐NC‐ND 4.0 license. (b) 1‐h workflow for the MPXV loop‐mediated isothermal amplification lateral flow biosensor (MPXV‐LAMP‐LFB) including the nucleic acid extraction, amplification, and result output steps. (c) Schematic representation of the MPXV‐LAMP‐LFB platform featuring the working principles of LAMP reactions (Clade I MPXV and Clade II MPXV) which leads to the formation of digoxin/biotin and FAM/biotin‐labeled amplicons post‐amplification and (d) the LFB and result output in which Clades I and II MPXV strains resulting in the T1 and T2 lines, respectively. CL acts as a control and appears if the result is negative and if one or both strains are present. Images (b–d) have been adapted from Reference [66] under Creative Commons CC‐BY license. LAMP, loop‐mediated isothermal amplification; LFB, lateral flow biosensor; MPXV, Mpox virus; VF‐RPA, vertical flow strip recombinase polymerase amplification.

In 2019, a method targeting the *G2R* gene using RPA was developed to detect MPXV Clades I and II.^60^ The RPA reaction took place at a single temperature of 42°C and had a sample‐answer readout time of 3–10 min. The assay had a high sensitivity and a limit of detection of 16 DNA molecules/μL. To measure the clinical success of this assay, 47 different sera and whole blood samples obtained from human samples and 48 different MPXV‐injected monkeys were tested. The MPXV‐RPA assay specificity value obtained at the end of this study was recorded as 100% (50/50). The sensitivity of the experiment was calculated as 95% (43/45). All stages of the experiment were carried out in a mobile bag laboratory powered by solar energy, demonstrating the feasibility of conducting RPA‐based studies with low‐tech equipment.^60^ In 2023, two different RPA methods, fluorescence‐RPA (F‐RPA) and vertical flow strip RPA (VF‐RPA), were developed by a group of researchers for use in MPXV detection.^63^ The F‐RPA method was performed on a small, portable fluorescence‐RPA device. Thus, the fluorescence curve could be followed in real time. The potential use of this platform in local medical units was suggested. VF‐RPA is another RPA method that requires a thermostatic bath. VF‐RPA results can be displayed on a strip, which facilitates the assay's use in PoC settings by offering features such as ease of use, sensitivity, and low cost. The VF‐RPA platforms appears promising as it can perform MPXV detection in field studies without the need for any other instrument and can be used quickly and reliably^63^ (Figure 3a). Despite its advantages, several limitations have to be overcome to achieve widespread adoption of RPA‐based devices for diagnosis. Some limitations include the fact that RPA‐based platforms can be contaminated by highly concentrated DNA, involves a rather laborious optimization process, and has limited flexibility provided by the kit formulation.

#### 3.2.3 Loop‐mediated isothermal amplification

LAMP is a molecular biology technique introduced in 2000.^89^ LAMP‐based pathogen detection is used to develop PoC tests, especially in the fight against infectious diseases.^90^ Among NAAT‐based techniques, LAMP method has an advantage over other techniques because it is faster, lower cost, and can be used in the field. Particularly during the COVID‐19 pandemic, researchers have focused on the LAMP technique to minimize the spread in countries with restricted laboratory resources.^91^ This amplification method can amplify the target nucleic acid with the Bst DNA polymerase enzyme at a constant temperature in 1 h.^92^ LAMP uses four sets of primers: two inner primers (forward inner primer [FIP] and backward inner primer [BIP]) specific to six different regions of the 130–300 bp target nucleic acid, and forward and backward outer primers (F3 and B3). The FIP contains F1c, which is the complementary to the F1 region of the target DNA, at the 5′ end and the F2 region of the target DNA at the 3′ end. The BIP contains B1c complementary to the B1 region of the target DNA at the 5′ end and the B2 region at the 3′ end. In this method, the two strands of the target nucleic acid are separated from each other by breaking hydrogen bonds by denaturation. The FIP primer binds to the F2c region of the strand in the 3′ → 5′ direction with its complement, the F2 region, and DNA polymerase synthesizes the opposite strand by adding complementary bases to the 3′ region of the FIP primer. The F3 primer binds to the upstream region of the binding site of the FIP primer, ensuring that the opposite strand of the target nucleic acid is fully synthesized by DNA polymerase. The strand synthesized with the FIP primer is separated from the target strand and forms a loop structure. A similar reaction is performed with primers B3 and BIP for the 5′ → 3′ strand of the target nucleic acid. The result is amplification products containing billions of inverted repeats of the target region and multi‐loop cauliflower‐like structures. Although not essential for the reaction, loop primers (LF and LB) complementary to the nucleic acid in the loop regions can be designed and included in the reaction to stabilize the loop structures and accelerate the reaction. Through the use of loop primers, reaction time can be reduced from 60 to 30 min.^90^, ^93^, ^94^, ^95^ Primers provide high specificity for amplification by recognizing six different sequences for amplification.

The most important stage in the development of the LAMP test for the detection of pathogenic microorganisms, especially DNA viruses, is primer design to ensure detection by amplifying the specific sequence of viral DNA with extremely high specificity using online tools.^96^, ^97^ In the PCR method, 1 million copies of the target sequence can be amplified using two primers (F, R), while in the LAMP method, 1 billion copies can be amplified using six primers.^98^ Compared to the PCR method, the LAMP amplification method has many advantages, such as not requiring expensive equipment, having higher sensitivity, and providing faster results.^98^ The Bst DNA polymerase enzyme isolated from Bacillus stearothermophilus plays a key role in LAMP reactions.^99^ This enzyme has strand‐switching activity and releases single‐stranded DNA during synthesis.^100^ Thus, the DNA denaturation step in PCR is bypassed. At the same time, the enzyme has a maximum effect at a constant temperature of 60‐65°C.^89^ Through this feature of the enzyme, the nucleic acid amplification process can be carried out under isothermal conditions without a time consuming cyclic reaction requiring thermal steps.^98^ The amplification process takes place at a constant temperature without the need for thermal cycling device commonly used in PCR.^98^ Thus, LAMP allows the development of PoC diagnostic platforms under isothermal conditions.^101^

A variety of methods are used to detect the amplification products obtained following the LAMP reaction.^90^ It is also possible to monitor amplification in real time. This real‐time monitoring is carried out with the real‐time PCR device and turbidimeter. The turbidimeter is preferred because it is more cost‐effective than the real‐time PCR device. Turbidimeter provides amplification product detection by measuring real‐time turbidity density. Magnesium pyrophosphate accumulated during the LAMP reaction creates a haze that can be seen with the naked eye. The turbidimeter device detects the amplified product in real time by reading the turbidity with 650 nm wavelength rays every 6 s. Various studies have used turbidimeters to detect desired microorganisms such as bacteria and parasites.^93^, ^102^ Agarose gel electrophoresis is another method used to observe amplification products in the LAMP method.^90^ To observe LAMP products under UV light, a 2% agarose gel is usually prepared and ladder‐like bands are observed due to the cauliflower‐like structure of LAMP products. However, using ethidium bromide, a carcinogenic chemical, in preparing agarose gel and opening the reaction tubes while loading the amplification products into the wells increases the risk of contamination. For these reasons, its use is limited. Additionally, product detection can be made under UV light by using fluorescent dyes such as SYBR Green I and calcein dye. Since HNB dye does not have fluorescent properties, the amplified product is detected by observing the color change with the naked eye. Lateral flow assay is another method used to detect LAMP products.^95^ It is preferred because it is low‐cost, easy to use, and portable. LAMP amplification products are conjugated with biotin, forming another complex with antibodies coated with gold nanoparticles. This complex moves along the test and control strips and detects the amplified product.^93^, ^102^, ^103^, ^104^

To achieve high‐accuracy and rapid diagnosis, it is very important to develop miniaturized PoC devices and platforms that do not require an expert to use. There have been several studies in the literature describing the development of PoC platforms disease diagnosis, especially for the SARS‐CoV‐2 virus.^105^, ^106^, ^107^, ^108^, ^109^, ^110^ These devices have a very high potential to control the spread of infectious diseases by offering rapid diagnosis. However, the number of studies conducted to detect the MPXV virus is quite limited.^111^ For example, researchers have developed a LAMP test to detect amplification of the MPXV virus using fluorescent calcein, observing turbidity with the naked eye.^64^ The *A27L* and *F3L* conserved regions of MPXV were chosen as the target sequence as they share low similarity with other orthopoxviruses. For LAMP amplification, a high specificity *A27L‐1* and *F3L‐1* primer set were designed and used on a real‐time turbidimeter for 60 min at an optimum temperature of 63°C. The samples containing the viral *A27L* and *F3L* genes were detected and the limit of detection was 20 copies/reaction. Turbidity values greater than 0.1 were accepted as positive results. Then, fluorescent calcein was added to the reaction mixtures, which were detected with the naked eye, and the change of color in the reaction mixture from orange to green was considered a positive result.^64^

In another study, Clades I and II were distinguished using a nanoparticle‐based biosensor using the LAMP method for MPX virus detection^66^ (Figure 3b–d). *D14L* genes of the Central African clade and *ATI* genes of the Clade II of the MPXV virus were selected as target sequences and specific LAMP primers were designed. LAMP reaction was prepared using two plasmids containing the *D14L* and *ATI* genes of Clade II MPXV. The reaction was carried out at 64°C for a total of 60 min, including virus detection analysis. MPXV detection was performed using real‐time turbidity, naked‐eye detection, and nanoparticle‐based lateral flow biosensor (LFB). When the turbidity value was greater than 0.1 and the reaction mixtures turned from orange to green with the naked eye, the test resulted was considered to be positive. For LFB detection, the 5′ ends of the FIP primers of the *D14L* and *ATI* genes were labeled with FAM and digoxin, and the LF primers of the two genes were labeled with biotin. LAMP‐LFB assay was performed using these newly designed labeled primers. Observation of a red stripe in both TL and CL regions of the LFB was considered a positive result. In the MPXV‐LAMP‐LFB method, the detection limit is stated as five copies for both *D14L* plasmid and *ATI* plasmid as a result of a 40‐minute reaction at 64°C.^66^

A novel procedure that can work with direct lesion samples that do not require DNA extraction for the rapid diagnosis of MPXV was described.^112^ Similarly, in another study, rapid and specific detection of the MPXV virus was performed using a lateral flow biosensor combined with the LAMP method by selecting the *ATI* gene target sequence.^65^ For the simultaneous detection of MPXV and Monkey B virus, the reaction was performed in 50 min on the PCR detection device using the HFman probe and the amplification curve was observed. The detection limit of MPXV was determined as 20 copies/reaction.^67^ In another study, the *N4R* gene was used for virus detection. Fluorescent LAMP reaction was prepared and the virus was detected with the amplification curve in the qPCR device at 65°C in 60 min. Additionally, a visual LAMP test was also performed. In the visual test using phenol red, a color change from pink to yellow was considered a positive result. The experiments were also conducted on clinical samples demonstrating the real‐life clinical feasibility of the platform.^68^

#### 3.2.4 Clustered regularly interspaced short palindromic repeats

The COVID‐19 epidemic has revealed the shortcomings of healthcare systems globally and specifically, countries that do not have fully equipped laboratory environments have had to face many critical problems in the diagnosis and follow‐up of the disease.^11^, ^12^ Due to the patient density during the COVID‐19 pandemic, healthcare systems became overwhelmed and various problems in PCR‐based diagnosis methods occurred globally, leaving healthcare almost inoperable.^11^ In order to address these problems, various PoC kits have been produced, and PoC approaches have become very important in the diagnosis of infectious diseases.^12^ One of the most valuable steps taken to overcome the limitations of traditional diagnostic methods is CRISPR‐based diagnostics technology.^40^ CRISPR‐associated (Cas)‐based detection, modeled on the adaptive immunity of prokaryotes, has led to a shift in infectious disease diagnosis from the traditional methods (such as PCR) to CRISPR‐based systems.^113^ One of the main advantages of CRISPR‐based diagnostic assays is that it can easily be integrated with PoC platforms. The ultimate goal in employing CRISPR‐based diagnostics systems is to create PoC tools where the non‐trained workers can accurately and rapidly diagnose infectious diseases in low‐resource settings.

**FIGURE 4:**
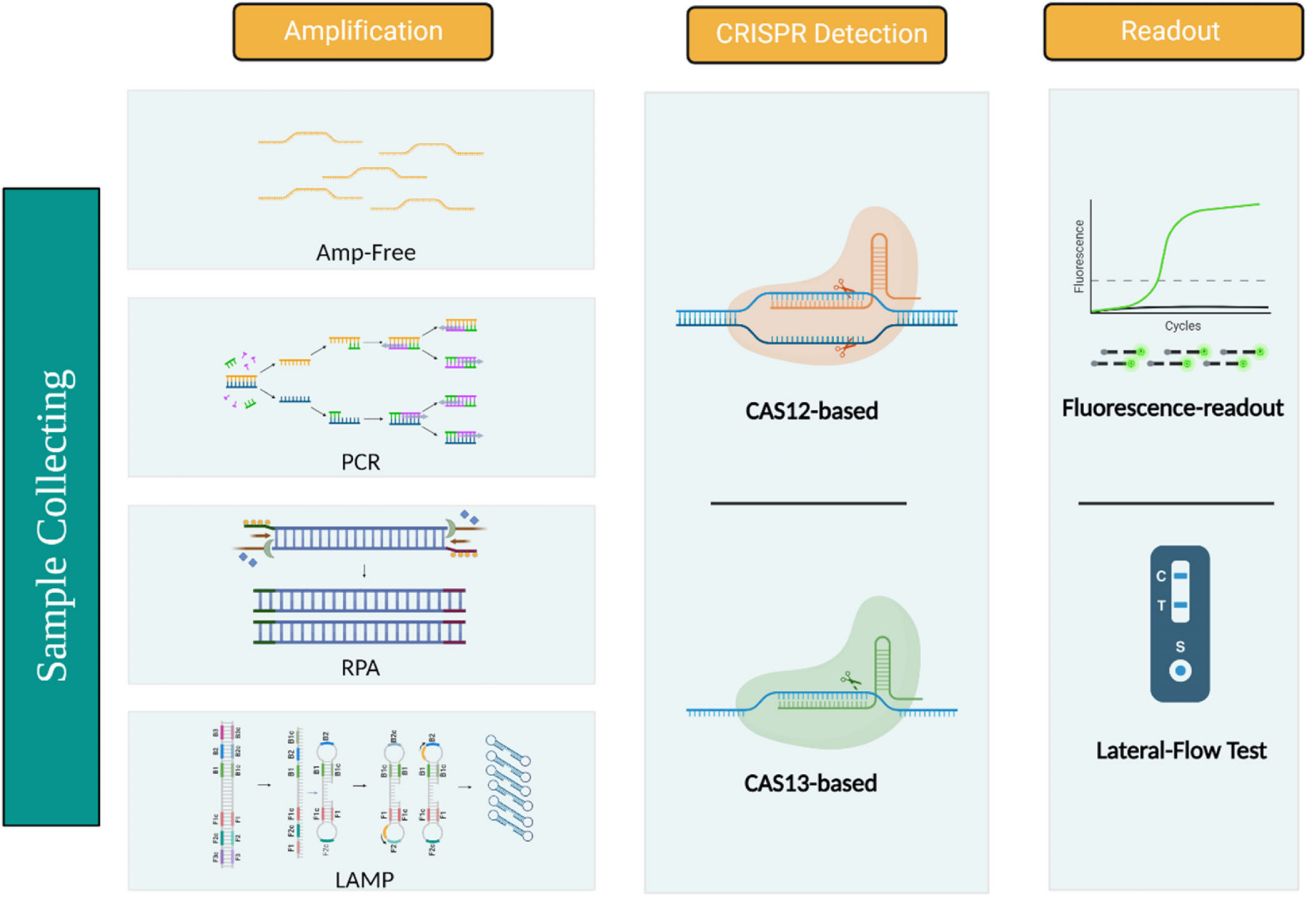
Schematic representation of next‐generation CRISPR combined detection technology. CRISPR‐based detection applications are basically divided into two; amplification‐free CRISPR and CRISPR with pre‐amplification. Traditional amplification methods such as PCR, isothermal amplification techniques such as RPA, and LAMP increase the assay's sensitivity by amplifying the viral product. Afterwards, viral nucleic acid is detected using CRISPR‐Cas systems 12 or 13. Finally, fluorescence‐readout and lateral‐flow test methods are used to observe and read the detection results. CRISPR, clustered regularly interspaced short palindromic repeats; LAMP, loop‐mediated isothermal amplification; PCR, polymerase chain reaction; RPA, recombinase polymerase amplification.

The Cas‐based detection method appears to be the most reliable tool in molecular diagnosis due to its single base specificity for the targeted genome, quick turnaround time, and atomic sensitivity level. Following the discovery of the CRISPR‐based programmable gene editing method, a significant increase in genetic research studies was observed globally.^113^ It is possible to categorize CRISPR‐based diagnostics methods under two groups: amplification‐free and amplification‐containing methods such as CRISPR combined with PCR, RPA, and LAMP (Figure 4). Amplification‐free diagnostics methods use members of the Cas enzyme family to perform direct detection of nucleic acids. Multiple targets are created to increase the detection signal, thus ensuring a more reliable viral detection. In diagnostic amplification methods, traditional amplification methods such as PCR or isothermal amplification methods such as RPA or LAMP are used for pre‐amplification of pathogen genome in samples followed by the use of Cas enzymes for the detection of the pre‐amplified genomic material. In these platforms, the result readouts are achieved using fluorescence or lateral flow assays and amplification‐free CRISPR systems can be designed using fluorescence readouts.^114^

The basic working principle of CRISPR‐based detection combines effector Cas proteins and CRISPR RNA (crRNA).^115^ The CRISPR‐based detection method proceeds under the guidance of crRNA, which is responsible for hybridizing the complementary sequence of the target DNA or RNA. In addition, Cas family proteins enable the recognition and cleavage of nucleic acids with high specificity.^116^ Over the years it has been demonstrated that different Cas family effectors, such as Cas9, Cas12, Cas13, and Cas14, can work for nucleotide detection with high efficiency, high sensitivity, and specificity.^114^

While Cas9 and Cas12 effectors are used in DNA manipulation, the Cas13 family can target RNA sequences.^117^ Additionally, Cas9 and Cas12a systems can be used against DNA and RNA viruses with DNA. Cas9, which has various limitations in targeting more than one gene at the same time, has high specificity in single targets, and is effective in targeting dsDNA.^117^ Cas12 and Cas13 systems have been created to eliminate such limitations of Cas9 systems. Cas13 provides a safe gene editing process by targeting mRNA instead of genomic DNA.^118^ Thus, Cas13 reduces undesirable off‐target effects. With the discovery of Cas12a's collateral cleavage activity, it became clear that it could be used to detect ssDNA without the need for any amplification process.^40^ This observed collateral cleavage activity may lead to signal amplification and thus enables measurement of ssDNA via irradiation.^119^ The concept of collateral crossover for nucleic acid‐targeting detection in pathogen diagnosis was first developed in 2016.^115^ The Specific High‐Sensitivity Enzymatic Reporter UnLOCKing (SHERLOCK) system significantly increased diagnostic sensitivity using CRISPR preceded by an amplification method.^120^ To simplify these diagnostic tools, early versions of CRISPR diagnostics used RPA for targeted sequence amplification.^86^ In later versions, RPA was replaced by the LAMP method,^40^ which offers higher specificity and sensitivity. Using isothermal amplification methods simplifies the technical processing and equipment requirements of CRISPR diagnostics, making it easier to transition to non‐hospital settings. Amplification‐free CRISPR detection techniques, using the targeting abilities of Cas effectors, may be very attractive for their simplicity. However, some significant drawbacks prevent the widespread use of these applications.^114^ In particular, a significant problem is the requirement for multiple target sites, which precludes the detection of single‐nucleotide polymorphisms and is dependent on sequence differences. Amplification‐free methods require RNA extraction to perform detection. This makes it difficult to perform such tests in situ and quickly before direct detection is achieved.^121^ Finally, the preferred nucleic acid type of Cas effectors significantly limits the flexibility of no‐amplification CRISPR, making it difficult to identify specific targets.^114^ To address this, researchers have focused on developing combined technologies by adding a two‐stage pre‐amplification process for CRISPR‐based diagnostic tools, and there are continued efforts to advance the technique at the PoC level.

In previous versions of CRISPR combined technologies, the main drawback of performing these assays in PoC settings was the transfer steps required in the 2‐step reaction. Significant progress has recently been made in the development of single‐reaction CRISPR combination technologies. A platform termed STOP (SHERLOCK one‐pot test) for the detection of SARS‐CoV‐2 was developed.^122^ For this assay, a thermostable Cas12 effector (AapCas12b) that showed strong crossover activity at high temperatures was identified.^123^ The thermostable property of this Cas12 enzyme made it possible to integrate this particular effector with LAMP in a single reaction. Another common challenge associated with combined CRISPR detection platforms is the nucleic acid isolation step, which requires preparation before the reaction. Therefore, this test used a simplified nucleic acid extraction protocol, allowing entire samples to be used as input, providing better detection than the CDC's gold standard qPCR protocol.

While CRISPR facilitates improvements in diagnostic technologies, it also allows for the development of new PoC devices. Taking advantage of the widespread use of smartphones as an opportunity, several CRISPR based diagnostic tools integrated with smartphones for easy readout have been developed. Two innovative methods using Cas13 and Cas12 were developed using the original SHERLOCK method and were termed SHINE and miSHERLOCK, respectively.^124^, ^125^ SHINE offers a more simplified detection method by improving the previously developed HUSDON^126^ lysis method and developing an app‐based fluorescence readout system to reduce sample contamination and the need for specialized personnel. On the other hand, miSHERLOCK aims to provide simplified lysis of viral particles and interpretation of results by developing a unique lysis method and device. This device is designed to allow users to automatically quantify results by monitoring them in a smartphone app, similar to the app in SHINE. As a different approach, another group succeeded in developing a smartphone‐compatible device in which many Cas12 detection reactions run simultaneously on one chip.^127^ This device is called CRISPR‐FDS and provides a user‐friendly experimental setup and interpretation, requiring no extraction steps and delivering rapid results with sensitivity comparable to qPCR. These innovations stand out as promising steps towards developing new devices or smartphone applications to simplify CRISPR diagnostic reactions and interpretation of results on PoC platforms. This points to a new generation of combined technologies coming into focus, helping to further optimize and facilitate the use of these PoC diagnostic tools.

**FIGURE 5:**
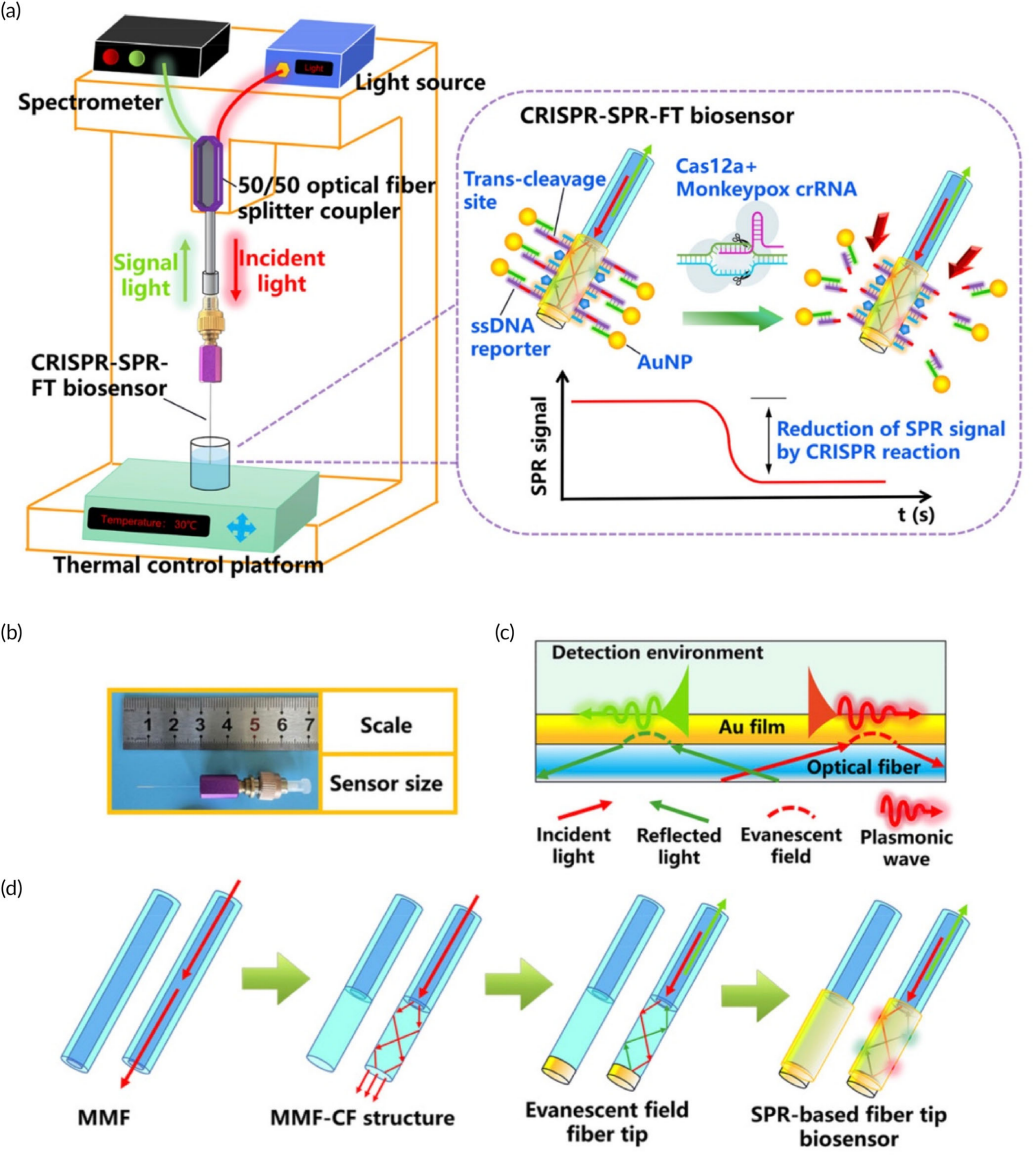
Portable CRISPR plasmon resonance‐based fiber type (CRISPR‐SPR‐FT) biosensing platform. (a) Schematic representation of the design. The portable device is composed of a biosensor. AuNPs with partial complementary DNAs and ssDNA reporters were used to detect MPXV. Cas12a‐crRNA is activated when target DNA is encountered, which leads to the trans‐cleavage of reporters at key sites, thereby leading to spectral signal transmission. A fiber‐based system is used to record signals in real‐time. (b) Photograph of the 125‐μm fiber tip of disposable SPR‐based biosensor. (c) SPR stimulation is depicted. (d) MMF and CF structures are illustrated. Adapted from Reference [70] under CC‐BY‐NC‐ND 4.0 license. CF, coreless fiber; CRISPR, clustered regularly interspaced short palindromic repeats; MMF, multimodal fiber; MPXV, Mpox virus; SPR, surface plasmon resonance.

In a study conducted in 2023, a next‐generation combined detection technology that combines CRISPR/Cas12a and RPA methods to detect MPXV Clades II and II was developed.^77^ Specific RPA primers were developed to target the *D14L* and *ATI* genes and were used in this new diagnostic CRISPR‐RPA protocol. RPA amplification products, whose activity was increased with the protospacer adjacent motif (PAM), achieved activation of the Cas12a effector by adhering to Cas12a/crRNA complexes. It has been stated that this effect also enables the single‐stranded DNA probe to undergo trans‐cleavage very quickly. In the assay performed, the limit of detection was 10 copies in each reaction for *D14L‐* and *ATI‐*plasmids. The platform had a total sample‐answer time of 45 min using the real‐time fluorescence method.^77^ In another study, a CRISPR/Cas12a‐based detection method with plasmon resonance‐based fiber type (CRISPR‐SPR‐FT) biosensor was designed in order to eliminate the problems in identifying asymptomatic and presymptomatic carrier patients^70^ (Figure 5). It has been stated that the CRISPR‐SPR‐FT biosensor with a diameter of 125 μm provides high stability and portability for MPXV diagnosis. Owing to the high specificity of this designed method, it is possible to make a definitive diagnosis of samples containing the critical mutation region *L108F* of the *F8L* gene. The CRISPR‐SPR‐FT system can detect viral dsDNA from the MPX virus in less than 1.5 h, without any amplification process. The limit of detection was below 5 aM in plasmids and 59.5 copies/μL in pseudovirus‐spiked blood samples.^70^ In another study on MPXV diagnosis, CRISPR‐Cas12a‐assisted nanopore (SCAN) was developed using RPA and CRISPR technologies.^76^ Although the SCAN method combined with RPA offers a sensitivity that cannot be achieved using SCAN alone, it enables POC applications that are vital for MPXV diagnosis, which PCR cannot provide due to its limitations. In MPXV‐specific RPA assays performed using the newly designed diagnosis method, the limit of detection was 19 copies in a 50 μL reaction. In addition, this kit could distinguish MPXV from cowpox virus with 100% accuracy in assays performed to measure its specificity in diagnosis. Owing to its miniaturization and programmability the potential application of the isothermal RPA‐SCAN device in PoC settings was also emphasized.^76^

In another study, a combined diagnostic technology called MASTR Pouch was developed for use in on‐site applications to overcome limitations of the PCR diagnostic method in PoC applications. By combining the CRISPR/Cas12a system and RPA, a method called MASTR Pouch that can perform MPXV diagnosis without the need for laboratories was presented.^75^ It was recorded that this palm‐sized sac completed the process from viral particle disintegration to easy readout with the naked eye in 35 min. The clinical sensitivity of this device, which performs fluorescence readout, was found to be 91.7%–95.8%. The device, which meets the ASSURED criteria established by WHO for PoC approaches, is an important product for MPXV diagnostics.^75^ MPXV Clades I and II were detected by developing a diagnostic system in which CRISPR technology was integrated into the LAMP method.^1^ LAMP amplification was carried out at 66°C for 60 min by targeting the *D14L* and *ATI* genes. MPXV‐LAMP amplification products nonspecifically cleaved single‐stranded DNA reporter molecules by binding to the target sequence under CRISPR/Cas12b gRNA guidance. CRISPR‐MPXV products were applied to the AuNP‐LFB detection system, and FAM and Biotin were separated from FAM and biotin‐labeled single‐stranded DNA probes and retained in the *CL* and *ATL* regions, respectively. Positive results were observed with a red stripe in both regions, and negative results were observed with a red stripe only in the *CL* region. The detection limit of the diagnostic system for the MPXV virus was determined as 10 copies/reaction.^1^

The role of PoC approaches in detecting MPXV are essential to the management of disease and control of the spread of infection. Many studies have been carried out to develop PoC tools for MPVX detection, most of which have used CRISPR/RPA‐LAMP‐based approaches. While these strategies have not yet been implemented on a widespread scale, they remain promising in enabling rapid, low‐cost, and accurate viral detection in low resource settings.

## 4 CONCLUSION

MPX is among the viral epidemics that prompted the WHO to declare a global public health emergency.^6^ While MPX has been endemic in the African region for several decades, it has recently become an outbreak threat globally. According to January 2024 data, the outbreak has spread to 117 countries.^28^ The number of patients suffering from viral infection is increasing by day. The reason for this is that transmission routes from person to person are very diverse and it often takes a long time to diagnose individuals with non‐specific prodromal clinical symptoms who are suspected of infection.

The qPCR method based on nucleic acid amplification, which is often considered the gold standard, is commonly used for the diagnosis of MPXV.^9^ However, the devices and equipment used for qPCR are quite costly. Additionally, the application of this method requires experts due to laborious protocols.^4^ It is very important to develop PoC diagnostic platforms that can be used in the field to control the spread of the disease in regions with limited resources. Studies on POC diagnostic platforms are required to control not only the MPXV outbreak but also to contain the spread of disease for other infective etiologies. In order to develop these diagnostic platforms, it is necessary to determine the optimum conditions for NAAT tests to obtain faster and more specific results and to integrate NAATs with technologies such as CRISPR/Cas that can increase assay sensitivity.

PoC diagnostic kits should be developed to be low‐cost, rapid, applicable in the field, highly sensitive, and not require an expert to use.^1^, ^8^ POC tests are also important to prevent transmission and control spread. To eliminate the need for non‐portable and high‐cost thermal cycler devices, platforms integrated with isothermal NAAT tests have been developed. In addition, platforms that can produce results easily and rapidly are underway.^1^ To this end, LAMP and RPA methods, which provide rapid nucleic acid amplification using specific primers at a constant temperature have been employed.^9^ The LAMP method provides amplification in 60 min under isothermal conditions of 60–65°C, using 4–6 primers designed specifically for the target gene sequence of the virus.^1^, ^9^ In the RPA method, rapid amplification is performed using recombinase, recombinase loading factor and single‐stranded binding protein at 37–43°C. When comparing LAMP with RPA, amplification products are obtained faster in RPA, but RPA is not sufficiently developed, and non‐specific amplicons frequently occur. Moreover, not fully optimizing primers and probes causes errors in RPA.^9^ The CRISPR/Cas system plays a fundamental role in the development of diagnostic platforms integrated with NAAT tests as it provides extra sensitivity and does not require expensive equipment. The basic principle of CRISPR/Cas diagnostic platforms is based on the trans‐cleavage activities of Cas enzymes, which can cleave the amplification products of Cas nucleases via guide RNA (gRNA).^1^, ^9^

MPXV has long been underestimated due to its low mortality and has been neglected from a global perspective. Its global spread should motivate research and development efforts to enable the rapid diagnosis of MPX at PoC. Thus, the diagnostic capacity should be improved and new studies should be added to the literature in order to prevent and control the spread of infection.

## AUTHOR CONTRIBUTIONS

**Nazente Atceken**: Conceptualization, Investigation, Funding acquisition, Writing – original draft, Methodology, Writing – review & editing, Visualization, Project administration, Supervision, Resources. **Ikra Bayaki**: Investigation, Writing – original draft, Visualization, Methodology, Conceptualization, Resources. **Berk Can**: Conceptualization, Investigation, Writing – original draft, Methodology, Visualization, Resources. **Defne Yigci**: Writing – review & editing, Visualization, Resources. **Savas Tasoglu**: Conceptualization, Investigation, Funding acquisition, Writing – original draft, Writing – review & editing, Visualization, Methodology, Project administration, Supervision, Resources.

## CONFLICT OF INTEREST STATEMENT

The authors declare no conflicts of interest.

## Data Availability

No new data were generated or analyzed in this study, and therefore, there are no data available for sharing.

## References

[1] Chen X , Yuan W , Yang X , et al. Ultrasensitive and specific identification of Monkeypox virus Congo basin and West African strains using a CRISPR/Cas12b‐based platform. Microbiol Spectr. 2023;11(2):e0403522. 36821485 10.1128/spectrum.04035-22 PMC10100855

[2] Jin B , Ma C , Zhang C , et al. Point‐of‐care detection of Monkeypox virus clades using high‐performance upconversion nanoparticle‐based lateral flow assay. Mikrochim Acta. 2024;191(4):177. 38441684 10.1007/s00604-024-06241-3

[3] Zahmatyar M , Fazlollahi A , Motamedi A , et al. Human monkeypox: history, presentations, transmission, epidemiology, diagnosis, treatment, and prevention. Front Med. 2023;10:1157670. 10.3389/fmed.2023.1157670 PMC10397518 37547598

[4] Yan H , Su JY , Tian L , et al. A rapid and sensitive fluorescent chromatography with cloud system for MPXV point‐of‐care diagnosis. Anal Chim Acta. 2024;1302:342514. 38580408 10.1016/j.aca.2024.342514

[5] Davis I , Payne JM , Olguin VL , et al. Development of a specific MPXV antigen detection immunodiagnostic assay. Front Microbiol. 2023;14:1243523. 10.3389/fmicb.2023.1243523 PMC10516133 37744911

[6] Huang Y , Mu L , Wang W . Monkeypox: epidemiology, pathogenesis, treatment and prevention. Signal Transduct Target Ther. 2022;7(1):373. 36319633 10.1038/s41392-022-01215-4 PMC9626568

[7] Pattnaik H , Surani S , Goyal L , Kashyap R . Making sense of Monkeypox: a comparison of other poxviruses to the Monkeypox. Cureus. 2023;15(4):e38083. 37252521 10.7759/cureus.38083 PMC10212748

[8] Stern D , Olson VA , Smith SK , et al. Rapid and sensitive point‐of‐care detection of Orthopoxviruses by ABICAP immunofiltration. Virol J. 2016;13(1):207. 27938377 10.1186/s12985-016-0665-5 PMC5148848

[9] Zhou Y , Chen Z . Mpox: a review of laboratory detection techniques. Arch Virol. 2023;168(8):221. 37543543 10.1007/s00705-023-05848-w PMC10404179

[10] Patel VM , Patel SV . Epidemiological review on monkeypox. Cureus. 2023;15(2):e34653. 36895541 10.7759/cureus.34653 PMC9991112

[11] Filip R , Gheorghita Puscaselu R , Anchidin‐Norocel L , Dimian M , Savage WK . Global challenges to public health care systems during the COVID‐19 pandemic: a review of pandemic measures and problems. J Pers Med. 2022;12(8):1295. 10.3390/jpm12081295 PMC9409667 36013244

[12] Wang C , Liu M , Wang Z , Li S , Deng Y , He N . Point‐of‐care diagnostics for infectious diseases: from methods to devices. Nano Today. 2021;37:101092. 33584847 10.1016/j.nantod.2021.101092 PMC7864790

[13] Hraib M , Jouni S , Albitar MM , Alaidi S , Alshehabi Z . The outbreak of monkeypox 2022: an overview. Ann Med Surg. 2022;79:104069. 10.1016/j.amsu.2022.104069 PMC9289401 35860140

[14] Likos AM , Sammons SA , Olson VA , et al. A tale of two clades: monkeypox viruses. J Gen Virol. 2005;86(Pt 10):2661‐2672. 16186219 10.1099/vir.0.81215-0

[15] Okwor T , Mbala PK , Evans DH , Kindrachuk J . A contemporary review of clade‐specific virological differences in monkeypox viruses. Clin Microbiol Infect. 2023;29(12):1502‐1507. 37507009 10.1016/j.cmi.2023.07.011

[16] Alakunle E , Moens U , Nchinda G , Okeke MI . Monkeypox virus in Nigeria: infection biology, epidemiology, and evolution. Viruses. 2020;12(11):1257. 10.3390/v12111257 PMC7694534 33167496

[17] Kugelman JR , Johnston SC , Mulembakani PM , et al. Genomic variability of monkeypox virus among humans, Democratic Republic of the Congo. Emerg Infect Dis. 2014;20(2):232‐239. 24457084 10.3201/eid2002.130118 PMC3901482

[18] Lum FM , Torres‐Ruesta A , Tay MZ , et al. Monkeypox: disease epidemiology, host immunity and clinical interventions. Nat Rev Immunol. 2022;22(10):597‐613. 36064780 10.1038/s41577-022-00775-4 PMC9443635

[19] von Magnus P , Andersen EK , Petersen KB , Birch‐Andersen A . A pox‐like disease in cynomolgus monkeys. Acta Pathol Microbiol Scand. 1959;46(2):156‐176.

[20] Ladnyj ID , Ziegler P , Kima E . A human infection caused by monkeypox virus in Basankusu territory, Democratic Republic of the Congo. Bull World Health Organ. 1972;46(5):593‐597. 4340218 PMC2480792

[21] Damon IK . Status of human monkeypox: clinical disease, epidemiology and research. Vaccine. 2011;29(suppl 4):D54‐D59. 22185831 10.1016/j.vaccine.2011.04.014

[22] Jezek Z , Gromyko AI , Szczeniowski MV . Human monkeypox. J Hyg Epidemiol Microbiol Immunol. 1983;27(1):13‐28. 6304185

[23] Reed KD , Melski JW , Graham MB , et al. The detection of monkeypox in humans in the Western hemisphere. N Engl J Med. 2004;350(4):342‐350. 14736926 10.1056/NEJMoa032299

[24] Yinka‐Ogunleye A , Aruna O , Ogoina D , et al. Reemergence of human Monkeypox in Nigeria, 2017. Emerg Infect Dis. 2018;24(6):1149‐1151. 29619921 10.3201/eid2406.180017 PMC6004876

[25] WHO . Multi‐country outbreak of mpox. External situation report 30. November 25. 2023 https://www.who.int/publications/m/item/multi-country-outbreak-of-mpox--external-situation-report-30---25-november-2023

[26] Lai CC , Hsu CK , Yen MY , Lee PI , Ko WC , Hsueh PR . Monkeypox: an emerging global threat during the COVID‐19 pandemic. J Microbiol Immunol Infect. 2022;55(5):787‐794. 35970757 10.1016/j.jmii.2022.07.004 PMC9352646

[27] WHO . Disease Outbreak News; Multi‐Country Monkeypox Outbreak in Non‐endemic Countries. World Health Organization; 2022. https://www.who.int/emergencies/disease-outbreak-news/item/2022-DON390

[28] WHO . 2022–23 Mpox Outbreak: Global Trends. World Health Organization; 2024. https://worldhealthorg.shinyapps.io/mpx_global/.

[29] WHO . Diagnostic Testing for the Monkeypox Virus (MPXV): Interim Guidance; World Health Organization. 2023. https://www.who.int/publications/i/item/who-mpx-laboratory-2023-1

[30] Mitjà O , Ogoina D , Titanji BK , et al. Monkeypox. Lancet. 2023;401(10370):60‐74. 36403582 10.1016/S0140-6736(22)02075-X PMC9671644

[31] Reynolds MG , Yorita KL , Kuehnert MJ , et al. Clinical manifestations of human monkeypox influenced by route of infection. J Infect Dis. 2006;194(6):773‐780. 16941343 10.1086/505880

[32] Altindis M , Puca E , Shapo L . Diagnosis of monkeypox virus—an overview. Travel Med Infect Dis. 2022;50:102459. 36109000 10.1016/j.tmaid.2022.102459 PMC9534096

[33] Hornuss D , Daehne T , Goetz V , et al. Transmission characteristics, replication patterns and clinical manifestations of human monkeypox virus‐an in‐depth analysis of four cases from Germany. Clin Microbiol Infect. 2023;29(1):112.e5‐112.e9. 10.1016/j.cmi.2022.09.012 PMC9534158 36155255

[34] Kisalu NK , Mokili JL . Toward understanding the outcomes of monkeypox infection in human pregnancy. J Infect Dis. 2017;216(7):795‐797. 29029238 10.1093/infdis/jix342 PMC6279131

[35] Mbala PK , Huggins JW , Riu‐Rovira T , et al. Maternal and fetal outcomes among pregnant women with human monkeypox infection in the Democratic Republic of Congo. J Infect Dis. 2017;216(7):824‐828. 29029147 10.1093/infdis/jix260

[36] McCollum AM , Damon IK . Human monkeypox. Clin Infect Dis. 2014;58(2):260‐267. 24158414 10.1093/cid/cit703 PMC5895105

[37] Patel A , Bilinska J , Tam JCH , et al. Clinical features and novel presentations of human monkeypox in a central London centre during the 2022 outbreak: descriptive case series. BMJ. 2022;378:e072410. 10.1136/bmj-2022-072410. 35902115 PMC9331915

[38] Singhal T , Kabra S , Lodha R . Monkeypox: a review. Indian J Pediatr. 2022;89(10):955‐960. 35947269 10.1007/s12098-022-04348-0 PMC9363855

[39] Yigci D , Atçeken N , Yetisen AK , Tasoglu S . Loop‐mediated isothermal amplification‐integrated CRISPR methods for infectious disease diagnosis at point of care. ACS Omega. 2023;8(46):43357‐43373. 38027359 10.1021/acsomega.3c04422 PMC10666231

[40] Atçeken N , Yigci D , Ozdalgic B , Tasoglu S . CRISPR‐Cas‐integrated LAMP. Biosensors. 2022;12(11):1035. 36421156 10.3390/bios12111035 PMC9688180

[41] Li M , Wang Y , Li C , et al. Development of monoclonal antibody‐based antigens detection assays for orthopoxvirus and monkeypox virus. J Infect. 2022;85(6):702‐769. 10.1016/j.jinf.2022.10.036 PMC9626448 36334727

[42] Chauhan RP , Fogel R , Limson J . Overview of diagnostic methods, disease prevalence and transmission of Mpox (formerly monkeypox) in humans and animal reservoirs. Microorganisms. 2023;11(5):1186. 10.3390/microorganisms11051186 PMC10223876 37317160

[43] Grossegesse M , Stern D , Hofmann N , et al. Serological methods for the detection of antibodies against monkeypox virus applicable for laboratories with different biosafety levels. J Med Virol. 2023;95(12):e29261. 38054557 10.1002/jmv.29261

[44] Yang X , Cheng X , Wei H , et al. Fluorescence‐enhanced dual signal lateral flow immunoassay for flexible and ultrasensitive detection of monkeypox virus. J Nanobiotechnol. 2023;21(1):450. 10.1186/s12951-023-02215-4 PMC10675944 38001482

[45] Liao H , Qu J , Lu H . Molecular and immunological diagnosis of Monkeypox virus in the clinical laboratory. Drug Discov Ther. 2022;16(6):300‐304. 36529507 10.5582/ddt.2022.01093

[46] Fowotade A , Fasuyi TO , Bakare RA . Re‐emergence of monkeypox in Nigeria: a cause for concern and public enlightenment. Afr J Clin Exp Microbiol. 2018;19(4):307.

[47] Weaver JR , Isaacs SN . Monkeypox virus and insights into its immunomodulatory proteins. Immunol Rev. 2008;225:96‐113. 18837778 10.1111/j.1600-065X.2008.00691.x PMC2567051

[48] Macneil A , Abel J , Reynolds MG , et al. Serologic evidence of human orthopoxvirus infections in Sierra Leone. BMC Res Notes. 2011;4:465. 22035219 10.1186/1756-0500-4-465 PMC3213095

[49] Karem KL , Reynolds M , Braden Z , et al. Characterization of acute‐phase humoral immunity to monkeypox: use of immunoglobulin M enzyme‐linked immunosorbent assay for detection of monkeypox infection during the 2003 North American outbreak. Clin Diagn Lab Immunol. 2005;12(7):867‐872. 16002637 10.1128/CDLI.12.7.867-872.2005 PMC1182207

[50] Colavita F , Mazzotta V , Rozera G , et al. Kinetics of viral DNA in body fluids and antibody response in patients with acute monkeypox virus infection. iScience. 2023;26(3):106102. 36748085 10.1016/j.isci.2023.106102 PMC9893533

[51] Green DM . Improving health care and laboratory medicine: the past, present, and future of molecular diagnostics. Proc (Bayl Univ Med Cent). 2005;18(2):125‐129. 16200160 10.1080/08998280.2005.11928050 PMC1200712

[52] Wang Y , Chen H , Lin K , et al. Ultrasensitive single‐step CRISPR detection of monkeypox virus in minutes with a vest‐pocket diagnostic device. Nat Commun. 2024;15(1):3279. 38627378 10.1038/s41467-024-47518-8 PMC11021474

[53] Schneider L , Blakely H , Tripathi A . Mathematical model to reduce loop mediated isothermal amplification (LAMP) false‐positive diagnosis. Electrophoresis. 2019;40(20):2706‐2717. 31206723 10.1002/elps.201900167 PMC7163742

[54] Hardinge P , Murray JA . Reduced false positives and improved reporting of loop‐mediated isothermal amplification using quenched fluorescent primers. Sci Rep. 2019;9(1):7400. 31089184 10.1038/s41598-019-43817-z PMC6517417

[55] Lillis L , Siverson J , Lee A , et al. Factors influencing recombinase polymerase amplification (RPA) assay outcomes at point of care. Mol Cell Probes. 2016;30(2):74‐78. 26854117 10.1016/j.mcp.2016.01.009 PMC4818709

[56] Li Y , Olson VA , Laue T , Laker MT , Damon IK . Detection of monkeypox virus with real‐time PCR assays. J Clin Virol. 2006;36(3):194‐203. 16731033 10.1016/j.jcv.2006.03.012 PMC9628957

[57] Li Y , Zhao H , Wilkins K , Hughes C , Damon IK . Real‐time PCR assays for the specific detection of monkeypox virus west African and Congo Basin strain DNA. J Virol Methods. 2010;169(1):223‐227. 20643162 10.1016/j.jviromet.2010.07.012 PMC9628942

[58] Mills MG , Juergens KB , Gov JP , et al. Evaluation and clinical validation of monkeypox (mpox) virus real‐time PCR assays. J Clin Virol. 2023;159:105373. 36603329 10.1016/j.jcv.2022.105373 PMC9783225

[59] Elbaz M , Halutz O , Ali Y , Adler A . Diagnosis of Monkeypox infection: validation of two diagnostic kits for viral detection using RT‐PCR. J Virol Methods. 2023;312:114653. 36395919 10.1016/j.jviromet.2022.114653 PMC9661602

[60] Davi SD , Kissenkötter J , Faye M , et al. Recombinase polymerase amplification assay for rapid detection of Monkeypox virus. Diagn Microbiol Infect Dis. 2019;95(1):41‐45. 31126795 10.1016/j.diagmicrobio.2019.03.015 PMC9629024

[61] Cui X , du B , Feng J , et al. Rapid detection of mpox virus using recombinase aided amplification assay. Front Cell Infect Microbiol. 2023;13:1008783. 36909721 10.3389/fcimb.2023.1008783 PMC9996015

[62] Gulinaizhaer A , Yang C , Zou M , Ma S , Fan X , Wu G . Detection of monkeypox virus using helicase dependent amplification and recombinase polymerase amplification combined with lateral flow test. Virol J. 2023;20(1):274. 37996921 10.1186/s12985-023-02223-8 PMC10668421

[63] Li Y , Gao Y , Tang Y , et al. Development of rapid nucleic acid assays based on the recombinant polymerase amplification for monkeypox virus. Virol Sin. 2023;38(1):165‐170. 36494078 10.1016/j.virs.2022.12.001 PMC9724568

[64] Feng J , Xue G , Cui X , et al. Development of a loop‐mediated isothermal amplification method for rapid and visual detection of monkeypox virus. Microbiol Spectr. 2022;10(5):e0271422. 36154444 10.1128/spectrum.02714-22 PMC9603857

[65] Huang X , Xiao F , Jia N , et al. Loop‐mediated isothermal amplification combined with lateral flow biosensor for rapid and sensitive detection of monkeypox virus. Front Public Health. 2023;11:1132896. 37033067 10.3389/fpubh.2023.1132896 PMC10080115

[66] Xiao F , Fu J , Huang X , et al. Loop‐mediated isothermal amplification coupled with nanoparticle‐based lateral flow biosensor for monkeypox virus detection. Talanta. 2024;269:125502. 38070288 10.1016/j.talanta.2023.125502

[67] Zeng Y , Zhao Y , Ren X , et al. Rapid detection of monkeypox virus and monkey B virus by a multiplex loop‐mediated isothermal amplification assay. J Infect. 2023;86(4):e114‐e116. 36792036 10.1016/j.jinf.2023.02.003 PMC9924052

[68] Yu C , Zuo L , Miao J , et al. Development of a novel loop‐mediated isothermal amplification method for the rapid detection of monkeypox virus infections. Viruses. 2022;15(1):84. 10.3390/v15010084 PMC9864920 36680124

[69] Iizuka I , Saijo M , Shiota T , et al. Loop‐mediated isothermal amplification‐based diagnostic assay for monkeypox virus infections. J Med Virol. 2009;81(6):1102‐1108. 19382264 10.1002/jmv.21494

[70] Chen Y , Chen Z , Li T , et al. Ultrasensitive and specific clustered regularly interspaced short palindromic repeats empowered a plasmonic fiber tip system for amplification‐free monkeypox virus detection and genotyping. ACS Nano. 2023;17(13):12903‐12914. 37384815 10.1021/acsnano.3c05007 PMC10340103

[71] Zhao F , Hu Y , Fan Z , et al. Rapid and sensitive one‐tube detection of mpox virus using RPA‐coupled CRISPR‐Cas12 assay. Cell Rep Methods. 2023;3(10):100620. 37848032 10.1016/j.crmeth.2023.100620 PMC10626268

[72] Low SJ , O'Neill MT , Kerry WJ , et al. Rapid detection of monkeypox virus using a CRISPR‐Cas12a mediated assay: a laboratory validation and evaluation study. Lancet Microbe. 2023;4(10):e800‐e810. 37722405 10.1016/S2666-5247(23)00148-9

[73] Gong L , Chen X , Wang Y , Liang J , Liu X , Wang Y . Rapid, sensitive, and highly specific detection of monkeypox virus by CRISPR‐based diagnostic platform. Front Public Health. 2023;11:1137968. 37441636 10.3389/fpubh.2023.1137968 PMC10335395

[74] Wang Y , Tang Y , Chen Y , et al. Ultrasensitive one‐pot detection of monkeypox virus with RPA and CRISPR in a sucrose‐aided multiphase aqueous system. Microbiol Spectr. 2024;12(1):e0226723. 38078721 10.1128/spectrum.02267-23 PMC10782985

[75] Wei J , Wang W , Yu Q , et al. MASTR pouch: palm‐size lab for point‐of‐care detection of Mpox using recombinase polymerase amplification and CRISPR technology. Sens Actuators B Chem. 2023;390:133950. 37193119 10.1016/j.snb.2023.133950 PMC10164292

[76] Ahamed MA , Khalid MAU , Dong M , et al. Sensitive and specific CRISPR‐Cas12a assisted nanopore with RPA for Monkeypox detection. Biosens Bioelectron. 2024;246:115866. 38029710 10.1016/j.bios.2023.115866 PMC10842690

[77] Yang X , Zeng X , Chen X , et al. Development of a CRISPR/Cas12a‐recombinase polymerase amplification assay for visual and highly specific identification of the Congo Basin and West African strains of mpox virus. J Med Virol. 2023;95(5):e28757. 37212293 10.1002/jmv.28757

[78] Chen Q , Gul I , Liu C , et al. CRISPR‐Cas12‐based field‐deployable system for rapid detection of synthetic DNA sequence of the monkeypox virus genome. J Med Virol. 2023;95(1):e28385. 36478250 10.1002/jmv.28385

[79] Singh M , Misra CS , Bindal G , Rangu SS , Rath D . CRISPR‐Cas12a assisted specific detection of mpox virus. J Med Virol. 2023;95(8):e28974. 37515526 10.1002/jmv.28974

[80] Mao L , Ying J , Selekon B , et al. Development and characterization of recombinase‐based isothermal amplification assays (RPA/RAA) for the rapid detection of monkeypox virus. Viruses. 2022;14(10):2112. 36298667 10.3390/v14102112 PMC9611073

[81] Michel J , Targosz A , Rinner T , et al. Evaluation of 11 commercially available PCR kits for the detection of monkeypox virus DNA, Berlin, July to September 2022. Euro Surveill. 2022;27(45):2200816. 10.2807/1560-7917.ES.2022.27.45.2200816 PMC9650706 36367010

[82] Papadakis G , Tran T , Druce J , Lim CK , Williamson DA , Jackson K . Evaluation of 16 molecular assays for the detection of orthopox and mpox viruses. J Clin Virol. 2023;161:105424. 36963141 10.1016/j.jcv.2023.105424 PMC10020139

[83] Paniz‐Mondolfi A , Guerra S , Muñoz M , et al. Evaluation and validation of an RT‐PCR assay for specific detection of monkeypox virus (MPXV). J Med Virol. 2023;95(1):e28247. 36271493 10.1002/jmv.28247

[84] Specchiarello E , Carletti F , Matusali G , et al. Development and validation of a nanoplate‐based digital PCR assay for absolute MPXV quantification. J Virol Methods. 2023;321:114802. 37625622 10.1016/j.jviromet.2023.114802

[85] Tan M , Liao C , Liang L , Yi X , Zhou Z , Wei G . Recent advances in recombinase polymerase amplification: principle, advantages, disadvantages and applications. Front Cell Infect Microbiol. 2022;12:1019071. 36519130 10.3389/fcimb.2022.1019071 PMC9742450

[86] Piepenburg O , Williams CH , Stemple DL , Armes NA . DNA detection using recombination proteins. PLoS Biol. 2006;4(7):e204. 16756388 10.1371/journal.pbio.0040204 PMC1475771

[87] Bai Y , Ji J , Ji F , et al. Recombinase polymerase amplification integrated with microfluidics for nucleic acid testing at point of care. Talanta. 2022;240:123209. 35026642 10.1016/j.talanta.2022.123209

[88] Feng X , Liu Y , Zhao Y , et al. Recombinase polymerase amplification‐based biosensors for rapid zoonoses screening. Int J Nanomed. 2023;18:6311‐6331. 10.2147/IJN.S434197 PMC10637217 37954459

[89] Notomi T , Okayama H , Masubuchi H , et al. Loop‐mediated isothermal amplification of DNA. Nucleic Acids Res. 2000;28(12):E63‐E663. 10871386 10.1093/nar/28.12.e63 PMC102748

[90] Atceken N , Munzer Alseed M , Dabbagh SR , Yetisen AK , Tasoglu S . Point‐of‐care diagnostic platforms for loop‐mediated isothermal amplification. Adv Eng Mater. 2023;25(8):2201174.

[91] Augustine R , Hasan A , das S , et al. Loop‐mediated isothermal amplification (LAMP): a rapid, sensitive, specific, and cost‐effective point‐of‐care test for coronaviruses in the context of COVID‐19 pandemic. Biology. 2020;9(8):182. 10.3390/biology9080182 PMC7464797 32707972

[92] Abbasi I , Kirstein OD , Hailu A , Warburg A . Optimization of loop‐mediated isothermal amplification (LAMP) assays for the detection of Leishmania DNA in human blood samples. Acta Trop. 2016;162:20‐26. 27288706 10.1016/j.actatropica.2016.06.009 PMC4987123

[93] Aoi Y , Hosogai M , Tsuneda S . Real‐time quantitative LAMP (loop‐mediated isothermal amplification of DNA) as a simple method for monitoring ammonia‐oxidizing bacteria. J Biotechnol. 2006;125(4):484‐491. 16790287 10.1016/j.jbiotec.2006.04.007

[94] Bhadra S , Ellington A . Portable nucleic acid tests for rapid detection of monkeypox virus. medRxiv. 2022:22278605.

[95] Parida M , Sannarangaiah S , Dash PK , Rao PVL , Morita K . Loop mediated isothermal amplification (LAMP): a new generation of innovative gene amplification technique; perspectives in clinical diagnosis of infectious diseases. Rev Med Virol. 2008;18(6):407‐421. 18716992 10.1002/rmv.593 PMC7169140

[96] Fu S , Qu G , Guo S , et al. Applications of loop‐mediated isothermal DNA amplification. Appl Biochem Biotechnol. 2011;163(7):845‐850. 20844984 10.1007/s12010-010-9088-8

[97] Li Y , Fan P , Zhou S , Zhang L . Loop‐mediated isothermal amplification (LAMP): a novel rapid detection platform for pathogens. Microb Pathog. 2017;107:54‐61. 28323152 10.1016/j.micpath.2017.03.016

[98] Soroka M , Wasowicz B , Rymaszewska A . Loop‐mediated isothermal amplification (LAMP): the better sibling of PCR? Cells. 2021;10(8):1931. 10.3390/cells10081931 PMC8393631 34440699

[99] Ma Y , Zhang B , Wang M , Ou Y , Wang J , Li S . Enhancement of polymerase activity of the large fragment in DNA polymerase I from *Geobacillus stearothermophilus* by site‐directed mutagenesis at the active site. Biomed Res Int. 2016;2016:2906484. 27981047 10.1155/2016/2906484 PMC5131239

[100] Nagamine K , Watanabe K , Ohtsuka K , Hase T , Notomi T . Loop‐mediated isothermal amplification reaction using a nondenatured template. Clin Chem. 2001;47(9):1742‐1743. 11514425

[101] Chaouch M . Loop‐mediated isothermal amplification (LAMP): an effective molecular point‐of‐care technique for the rapid diagnosis of coronavirus SARS‐CoV‐2. Rev Med Virol. 2021;31(6):e2215. 33476080 10.1002/rmv.2215 PMC7995099

[102] Aonuma H , Yoshimura A , Kobayashi T , et al. A single fluorescence‐based LAMP reaction for identifying multiple parasites in mosquitoes. Exp Parasitol. 2010;125(2):179‐183. 20064511 10.1016/j.exppara.2009.12.023

[103] Chen C , Zhao Q , Guo J , Li Y , Chen Q . Identification of methicillin‐resistant *Staphylococcus aureus* (MRSA) using simultaneous detection of mecA, nuc, and femB by loop‐mediated isothermal amplification (LAMP). Curr Microbiol. 2017;74(8):965‐971. 28573341 10.1007/s00284-017-1274-2

[104] Wong YP , Othman S , Lau YL , Radu S , Chee HY . Loop‐mediated isothermal amplification (LAMP): a versatile technique for detection of micro‐organisms. J Appl Microbiol. 2018;124(3):626‐643. 29165905 10.1111/jam.13647 PMC7167136

[105] Ayankojo AG , Boroznjak R , Reut J , Öpik A , Syritski V . Molecularly imprinted polymer based electrochemical sensor for quantitative detection of SARS‐CoV‐2 spike protein. Sens Actuators B Chem. 2022;353:131160. 34866797 10.1016/j.snb.2021.131160 PMC8626155

[106] Eissa S , Zourob M . Development of a low‐cost cotton‐tipped electrochemical immunosensor for the detection of SARS‐CoV‐2. Anal Chem. 2021;93(3):1826‐1833. 33370087 10.1021/acs.analchem.0c04719

[107] Fabiani L , Saroglia M , Galatà G , et al. Magnetic beads combined with carbon black‐based screen‐printed electrodes for COVID‐19: a reliable and miniaturized electrochemical immunosensor for SARS‐CoV‐2 detection in saliva. Biosens Bioelectron. 2021;171:112686. 33086175 10.1016/j.bios.2020.112686 PMC7833515

[108] Hussein HA , Kandeil A , Gomaa M , Mohamed el Nashar R , el‐Sherbiny IM , Hassan RYA . SARS‐CoV‐2‐impedimetric biosensor: virus‐imprinted chips for early and rapid diagnosis. ACS Sens. 2021;6(11):4098‐4107. 34757734 10.1021/acssensors.1c01614

[109] Raziq A , Kidakova A , Boroznjak R , Reut J , Öpik A , Syritski V . Development of a portable MIP‐based electrochemical sensor for detection of SARS‐CoV‐2 antigen. Biosens Bioelectron. 2021;178:113029. 33515985 10.1016/j.bios.2021.113029 PMC7826012

[110] Stefano JS , Guterres e Silva LR , Rocha RG , et al. New conductive filament ready‐to‐use for 3D‐printing electrochemical (bio)sensors: towards the detection of SARS‐CoV‐2. Anal Chim Acta. 2022;1191:339372. 35033268 10.1016/j.aca.2021.339372 PMC9381826

[111] Stefano JS , Silva LRG , Kalinke C , et al. Human monkeypox virus: detection methods and perspectives for diagnostics. TrAC Trends Anal Chem. 2023;167:117226.

[112] Li Z , Sinha A , Zhang Y , et al. Extraction‐free LAMP assays for generic detection of Old World Orthopoxviruses and specific detection of Mpox virus. Sci Rep. 2023;13(1):21093. 38036581 10.1038/s41598-023-48391-z PMC10689478

[113] Li H , Xie Y , Chen F , et al. Amplification‐free CRISPR/Cas detection technology: challenges, strategies, and perspectives. Chem Soc Rev. 2023;52:361‐382. doi:10.1039/d2cs00594h 36533412

[114] Brogan DJ , Akbari OS . CRISPR diagnostics: advances toward the point of care. Biochemistry. 2023;62(24):3488‐3492. 35325535 10.1021/acs.biochem.2c00051 PMC10564243

[115] East‐Seletsky A , O'Connell MR , Knight SC , et al. Two distinct RNase activities of CRISPR‐C2c2 enable guide‐RNA processing and RNA detection. Nature. 2016;538(7624):270‐273. 27669025 10.1038/nature19802 PMC5576363

[116] Huang T , Zhang R , Li J . CRISPR‐Cas‐based techniques for pathogen detection: retrospect, recent advances, and future perspectives. J Adv Res. 2023;50:69‐82. 36367481 10.1016/j.jare.2022.10.011 PMC10403697

[117] Escalona‐Noguero C , López‐Valls M , Sot B . CRISPR/Cas technology as a promising weapon to combat viral infections. Bioessays. 2021;43(4):e2000315. 33569817 10.1002/bies.202000315 PMC7995209

[118] Bigini F , Lee SH , Sun YJ , Sun Y , Mahajan VB . Unleashing the potential of CRISPR multiplexing: harnessing Cas12 and Cas13 for precise gene modulation in eye diseases. Vision Res. 2023;213:108317. 37722240 10.1016/j.visres.2023.108317 PMC10685911

[119] van Dongen JE , Berendsen JTW , Steenbergen RDM , Wolthuis RMF , Eijkel JCT , Segerink LI . Point‐of‐care CRISPR/Cas nucleic acid detection: recent advances, challenges and opportunities. Biosens Bioelectron. 2020;166:112445. 32758911 10.1016/j.bios.2020.112445 PMC7382963

[120] Gootenberg JS , Abudayyeh OO , Lee JW , et al. Nucleic acid detection with CRISPR‐Cas13a/C2c2. Science. 2017;356(6336):438‐442. 28408723 10.1126/science.aam9321 PMC5526198

[121] Ramachandran A , Santiago JG . CRISPR enzyme kinetics for molecular diagnostics. Anal Chem. 2021;93(20):7456‐7464. 33979119 10.1021/acs.analchem.1c00525

[122] Joung J , Ladha A , Saito M , et al. Detection of SARS‐CoV‐2 with SHERLOCK one‐pot testing. N Engl J Med. 2020;383(15):1492‐1494. 32937062 10.1056/NEJMc2026172 PMC7510942

[123] Teng F , Cui T , Feng G , et al. Repurposing CRISPR‐Cas12b for mammalian genome engineering. Cell Discov. 2018;4:63. 30510770 10.1038/s41421-018-0069-3 PMC6255809

[124] Arizti‐Sanz J , Freije CA , Stanton AC , et al. Streamlined inactivation, amplification, and Cas13‐based detection of SARS‐CoV‐2. Nat Commun. 2020;11(1):5921. 33219225 10.1038/s41467-020-19097-x PMC7680145

[125] de Puig H , Lee RA , Najjar D , et al. Minimally instrumented SHERLOCK (miSHERLOCK) for CRISPR‐based point‐of‐care diagnosis of SARS‐CoV‐2 and emerging variants. Sci Adv. 2021;7(32):2944. 10.1126/sciadv.abh2944 PMC8346217 34362739

[126] Myhrvold C , Freije CA , Gootenberg JS , et al. Field‐deployable viral diagnostics using CRISPR‐Cas13. Science. 2018;360(6387):444‐448. 29700266 10.1126/science.aas8836 PMC6197056

[127] Ning B , Yu T , Zhang S , et al. A smartphone‐read ultrasensitive and quantitative saliva test for COVID‐19. Sci Adv. 2021;7(2):3703. 10.1126/sciadv.abe3703 PMC7793573 33310733

